# Synthesis, Anticancer Activity, and Docking Studies of Novel Hydroquinone-Chalcone-Pyrazoline Hybrid Derivatives

**DOI:** 10.3390/ijms25137281

**Published:** 2024-07-02

**Authors:** Javier Maldonado, Alfonso Oliva, Leda Guzmán, Aurora Molinari, Waldo Acevedo

**Affiliations:** Instituto de Química, Facultad de Ciencias, Pontificia Universidad Católica de Valparaíso, Valparaíso 23732223, Chile; javier.maldonado@pucv.cl (J.M.); alfonso.oliva@pucv.cl (A.O.); leda.guzman@pucv.cl (L.G.)

**Keywords:** pyrazoline-1,4-benzohydroquinone, antiproliferative activity, molecular docking

## Abstract

A novel series of antitumor hybrids was synthesized using 1,4-benzohydroquinone and chalcone, furane, or pyrazoline scaffolds. This were achieved through isosteric substitution of the aryl group of the chalcone β-carbon with the furanyl moiety and structural modification of the α,β-unsaturated carbonyl system. The potential antitumor activity of these hybrids was evaluated in vivo on MCF-7 breast adenocarcinoma and HT-29 colorectal carcinoma cells, demonstrating cytotoxic activity with IC_50_ values ranging from 28.8 to 124.6 µM. The incorporation of furan and pyrazoline groups significantly enhanced antiproliferative properties compared to their analogues and precursors (**VII**–**X**), which were inactive against both neoplastic cell lines. Compounds **4**, **5**, and **6** exhibited enhanced cytotoxicity against both cell lines, whereas compound 8 showed higher cytotoxic activity against HT-29 cells. Molecular docking studies revealed superior free-energy values (ΔG*_bin_*) for carcinogenic pathway-involved kinase proteins, with our in silico data suggesting that these derivatives could be promising chemotherapeutic agents targeting kinase pathways. Among all the synthesized PIBHQ compounds, derivatives **7** and **8** exhibited the best drug-likeness properties, with values of 0.53 and 0.83, respectively. ADME results collectively suggest that most of these compounds hold promise as potential candidates for preclinical assays.

## 1. Introduction

Cancer results from uncontrolled cell division caused by deregulation of the biological mechanisms controlling the cell cycle [1]. The sustained increases in cancer incidence and mortality over the last decade necessitate the discovery of new compounds with superior anticancer properties compared to current chemotherapeutic drugs [2].

Hydroquinone represents a simple phenolic base structure found as secondary metabolites in various natural products and serves as a privileged scaffold in bioorganic and medicinal chemistry [3,4,5,6]. These compounds exhibit a wide range of biological activities, including antineoplastic, anti-inflammatory, antileishmanial, antimicrobial, and antioxidant properties [4,7,8,9]. Several hydroquinones have demonstrated antitumor activity against breast, colon, lung, and cervical cancers, among others [7,10,11]. Their antineoplastic properties are primarily attributed to their ability to generate reactive oxygen species (ROS) and induce significant DNA damage [12,13]. Specifically, alkyl hydroquinones have been implicated in antiproliferative processes through various mechanisms, such as downregulation of p53, inhibition of protein kinases, tubulin polymerization, and cyclooxygenases [8,9,14,15,16].

Chalcones are structures of both natural and synthetic origin that exhibit various pharmacological activities, including antineoplastic, antioxidant, anti-inflammatory, antiviral, antimalarial, antimicrobial, analgesic, and antitubercular properties [17,18,19,20]. Studies on their anticancer properties have shown that they interact with various target proteins involved in proliferative processes. One of the most recognized antineoplastic mechanisms of these derivatives is the interruption of the cell cycle through microtubule disruption by binding to tubulins, preventing their polymerization. This disruption in mitotic spindle assembly and cytoskeletal function results in cell cycle arrest in the G2/M phase [21]. Additionally, chalcones can activate p53 protein; inhibit topoisomerase activity; halt angiogenesis by inhibiting vascular endothelial growth factor (VEGF); and inhibit various enzymes such as androgen receptor, kinases, mesenchymal–epithelial transition factor (MET), cyclooxygenase 2 (COX-2), histone deacetylases (HDACs), nuclear factor kappa B (NF-κB), and epidermal growth factor receptor (EGFR). These biological processes collectively induce apoptosis [22,23,24].

Chalcones containing aromatic heterocyclic groups in their structure enhance the aforementioned biological activities [25,26]. In particular, the furan ring in chalcone structures has shown significant anticancer properties [20,21,27,28]. Studies have indicated that the bioisosteric exchange of the aryl group for this aromatic heterocycle in chalcones leads to the synthesis of potent inhibitors of protein tyrosine phosphatase 1B (PTP1B) [29].

Pyrazoline is a five-membered heterocycle containing two nitrogen atoms and holds great pharmacological interest. This versatile scaffold is used to develop new anticancer agents, and many clinically used anticancer drugs contain this group [30]. Specifically, pyrazoline derivatives have been involved in antineoplastic processes through inhibition of VEGFR-2, EGFR, tubulin polymerization deregulation, and inhibition of topoisomerase II. These processes inhibit cell division, DNA synthesis, or cell migration [31,32,33,34].

**Figure 1:**
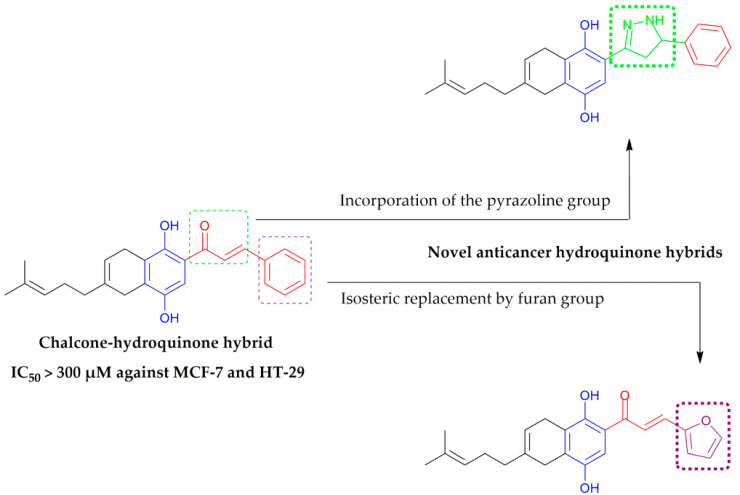
Design of novel hydroquinone hybrids as anticancer agents.

Molecular hybridization is a strategy in medicinal chemistry to discover new compounds with pharmacological properties by combining two or more structural units with recognized biological activities into a single structure [22,35,36]. In previous studies, we synthesized a series of hybrid derivatives of naphthoquinones conjugated with isoxazole [37,38] and pyrazole [39,40], as well as chalcones fused to naphthoquinone/hydroquinone systems with promising antineoplastic properties [41]. In this work, we rationally designed and synthesized a new series of chalcone–hydroquinone hybrids, including structural modifications of compounds previously prepared by our research group [41]. We performed an isosteric replacement of the aryl group of the chalcone β-carbon with the furanyl heterocycle. Additionally, by modifying the α,β-unsaturated carbonyl system, we synthesized novel pyrazoline-1,4-benzohydroquinone (PIBHQ) hybrids (Figure 1). Finally, we assessed the in vitro antineoplastic activity of these hybrids on MCF-7 and HT-29 cancer cell lines and conducted in silico molecular docking studies to identify potential biological targets through virtual screening. This included various cancer-related proteins overexpressed in breast and colon cancers to evaluate the antitumor potential and explore the possible action mechanisms of these new hybrid compounds.

## 2. Results and Discussion

### 2.1. Chemistry

**Scheme 1:**
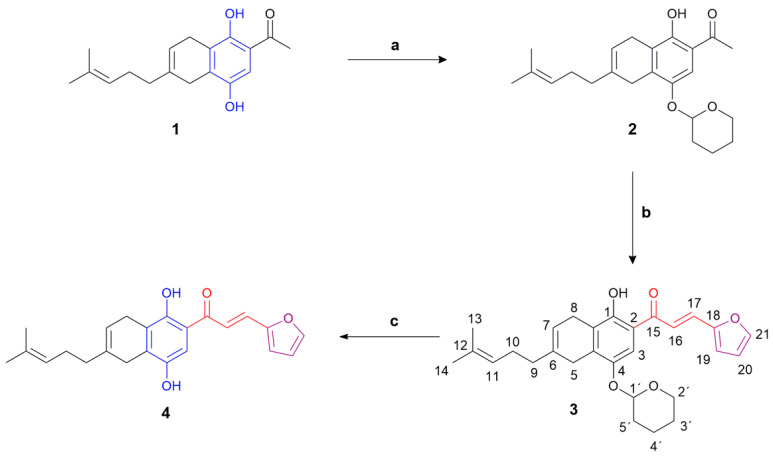
Synthesis route of hybrid **4**. Reagents and conditions: (a) DHP, PPTS, CH_2_Cl_2_, rt, 19 h; (b) Ba(OH)_2_·8H_2_O, 2-furaldehyde, EtOH, 90 °C, 30 min; (c) PTSA, MeOH, rt, 3 h.

As shown in Scheme 1, the synthesis of compounds **3** and **4** was derived from the precursor 1-{1-hydroxy-6-(4-methylpent-3-en-1-yl)-4-[(tetrahydro-2H-pyran-2-yl)oxy]-5,8-dihydronaphthalen-2-yl}ethanone naphthohydroquinone (**2**). Initially, the phenolic -OH group attached to C-4 of compound **1** was protected using 3,4-dihydro-2H-pyran (DHP) in the presence of pyridinium p-toluenesulfonate (PPTS) as a catalyst according to our previously described procedure [41]. This step was necessary to eliminate the acidic properties of the phenolic group.

The new hybrid, chalcone/1,4-benzohydroquinone (CBHQ) (**4**), was synthesized via Claisen–Schmidt condensation of precursor **2** with 2-furfuraldehyde, yielding derivative **3** with a moderate yield of 63%. Subsequent acid hydrolysis using 4-toluenesulfonic acid monohydrate (PTSA) allowed for the high-yield (92%) production of CBHQ **4**.

The structures of all new compounds were determined by IR, ^1^H NMR (400 MHz), ^13^C NMR (100 MHz), and elemental analysis. The infrared (IR) spectra of derivatives **3** and **4** exhibited characteristic absorption bands of the stretching vibration (*stv*) of the phenolic O-H bonds in the range of 3374–3481 cm^−1^, in addition to the *stv* bands of the C=O bonds at 1634–1639 cm^−1^. The *stv* band of C=C bond of chalcones was observed in the range 1581–1583 cm^−1^. Moreover, the ^1^H NMR spectrum of compound **3** showed signals corresponding to the protons of the α,β-unsaturated fragment characteristic of H-16 and H-17 chalcones at δ 7.50 and δ 7.68 ppm, respectively. The analogous protons H-16 and H-17 of CBHQ **4** were observed at δ 7.45 and δ 7.65 ppm, respectively, with both protons of the unsaturated system showing a coupling constant (*J*) of 15.1 Hz. Phenolic protons 4-OH and 1-OH appeared as singlets at δ 4.80 and 13.00 ppm, respectively. This marked difference in chemical shift is due to the intramolecular interaction of the 1-OH proton with the carbonyl group of the chalcone fragment. Additionally, the signals for protons H-20, H-19, and H-21 of the furan heterocycle were observed at δ 6.54, 6.74, and 7.54 ppm, respectively.

In the ^13^C NMR spectrum of CBHQ **4**, signals attributed to all carbons of the α,β-unsaturated carbonyl fragment characteristic of chalcones were observed. Particularly, the carbonyl at C-15 absorbed at δ 192.5 ppm, while the signals of carbons C-20, C-19, C-21, and C-18 of the furan heterocycle appeared at δ 112.8, 116.8, 145.3, and 151.6 ppm, respectively. The IR, ^1^H NMR, and ^13^C NMR spectra of compounds **3** and **4** are presented in Figures S1–S6.

**Scheme 2:**
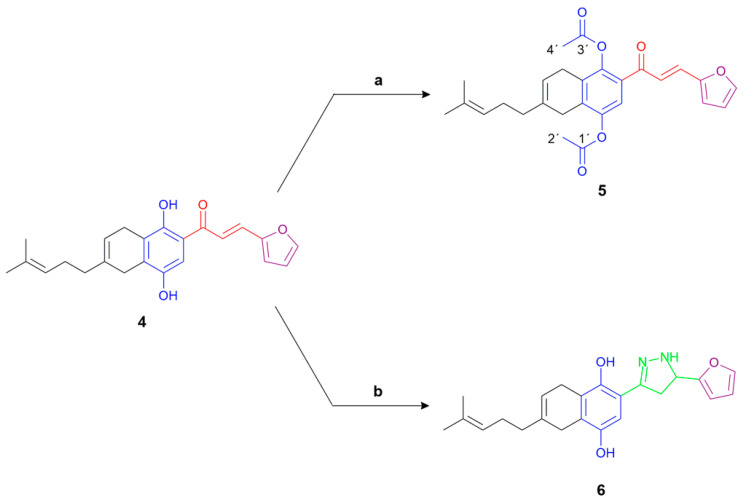
Synthesis of hybrid molecules **5** and **6**. Reagents and conditions: (a) acetic anhydride, pyridine, rt, 24 h; (b) N_2_H_4_·H_2_O, EtOH, reflux, 5 h.

As shown in Scheme 2, the synthesized CBHQ **4** facilitated the production of new compounds **5** and **6**. For the synthesis of CBHQ **5**, acetylation of the phenolic groups of CBHQ **4** was performed using acetic anhydride in pyridine, achieving a high yield of 96%. Additionally, the reactivity of the α, β-unsaturated carbonyl system of CBHQ **4** was exploited to carry out a conjugated addition with hydrazine, resulting in the hybrid PIBHQ **6** containing the pyrazoline heterocycle. This synthesis utilized excess hydrazine monohydrate in ethanol under reflux, yielding a product with a 71% yield (Scheme 2).

The infrared (IR) spectrum of acetylated CBHQ **5** showed an *stv* absorption band of the C=O of the acetyl groups at 1766 cm^−1^. The ^1^H NMR spectrum exhibited a multiplet signal corresponding to the protons of acetyl groups H-2′ and H-4′ at δ 2.26 ppm, while the signals of the H-16 and H-17 protons appeared as doublets at δ 7.10 and δ 7.43 ppm, respectively, with coupling constants (*J*) of 15.5 Hz. In the ^13^C spectrum, the signals corresponding to methyls C-2′ and C-4′ of the acetyl groups appeared at δ 20.8 and 20.9 ppm, respectively, while intense signals of both carbonyls of the C-3′ and C-1′ acetyl groups were observed at δ 169.0 and 169.1 ppm, respectively.

Regarding the structural characterization of PIBHQ **6**, the IR spectrum displayed an *stv* band at 3328 cm^−1^ of the phenolic O-H bond and a N-H bond of pyrazoline, while the *stv* band of the C=N bond was observed at 3328 cm^−1^. The ^1^H NMR spectrum showed the signals corresponding to protons H-16, H-17, H-19, H-20, and H-21 at δ 3.29, 4.88, 6.23, 6.32, and 7.37 ppm, respectively. In the ^13^C NMR spectrum, signals C-16, C-17, C-19, C-20, and C-21 were observed at δ 37.9, 55.5, 106.4, 110.3, and 142.5 ppm, respectively. The C-15 signal of the pyrazoline heterocycle was also observed at a higher chemical shift to δ 155.1 ppm. The IR, ^1^H NMR, and ^13^C NMR spectra of compounds **5** and **6** are shown in Figures S7–S12.

**Scheme 3:**
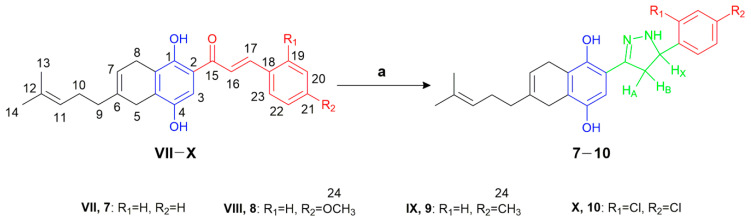
Synthesis of hybrid molecules **7**, **8**, **9**, and **10**. Reagents and conditions: (a) N_2_H_4_·H_2_O, EtOH, reflux, 5 h.

Utilizing the structural similarity of compounds **VII**–**X** synthesized in previous studies [41] with the hybrid CBHQ **4**, we proposed the synthesis of new hybrid molecules of pyrazoline-type PIBHQs (**7**–**10**). This was achieved through a cyclocondensation reaction of the α,β-unsaturated carbonyl fragment of precursors **VII**–**X** with hydrazine monohydrate under reflux heating in ethanol as a solvent (Scheme 3). PIBHQs **7**–**10** were obtained with moderate yields (62–75%), with higher yields observed for the 2,4-dichloro substituents in the phenyl group.

For the ^1^H NMR characterization of PIBHQs **7**–**10**, the elucidation of the chemical skeleton of the pyrazolines focused on analyzing the signals corresponding to the *ABX* hydrogen system of the heterocyclic ring. The ^1^H NMR spectra showed the signal of the H-16_A_ proton in the range δ 2.84–3.06 ppm, while the H-16_B_ proton signal appeared between δ 3.36 and 3.61 ppm. The H-17_X_ proton signal was observed in the range of δ 4.79–5.18 ppm. Generally, the magnetically non-equivalent diastereotopic protons of the methylene group H-16_A_ and H-16_B_ couple with the H-17_X_ proton displayed doublet–doublet signals with geminal and vicinal coupling constants (*J*_AB_ = 16.3–16.7 Hz, *J*_AX_ = 8.1–9.4 Hz and *J*_BX_ = 10.2–10.7 Hz). The IR, ^1^H NMR, and ^13^C NMR spectra of compounds **7**–**10** are shown in Figures S13–S24.

### 2.2. In Vitro Cytotoxicity Assays

The antiproliferative activity of the new compounds (**4**–**10**) assessed on MCF-7 and HT-29 cancer cell lines showed a cytotoxic effect due to the conjugation of chalcone and pyrazoline pharmacophores with the hydroquinone derivatives, a we previously evaluated in these cell cultures, which are among the most common types of cancer, as previously evaluated on cancer cell lines. The results are shown in Table 1 and are expressed as the half-maximal inhibitory concentration (IC_50_ ± standard deviation) of cell proliferation for each evaluated compound, along with the calculated −log_10_ of that value (pIC_50_).

As presented in Table 1, CBHQs **4** and **5** and PIBHQ **6** hybrids containing a furan ring exhibited a notable cytotoxic effect against both cancer cell lines. For MCF-7, the pIC_50_ values exceeded 4.46, while for HT-29, the pIC_50_ values were >4.42. CBHQ **5** demonstrated the highest cytotoxicity, with pIC_50_ values of 4.73 and 4.66 for HT-29 and MCF-7, respectively. These results indicate that diacetylation in CBHQ hybrids enhances cytotoxicity against both neoplastic cell lines, consistent with previously reported studies [41].

Cytotoxic activity of CBHQ hybrids **VII**–**X** from our previous studies was compared with that of the analogous compounds synthesized in this work, which involved the isosteric substitution of the aryl ring with the furanyl heterocycle in the chalcone moiety. The results showed that CBHQ 4 exhibited significant cytotoxicity against MCF-7 and HT-29 cell lines, with pIC_50_ values of 4.54 and 4.47, respectively, notably enhancing the antiproliferative properties compared to their **VII**–**X** analogues, which were inactive against both tested neoplastic cell lines [41].

**Figure 2:**
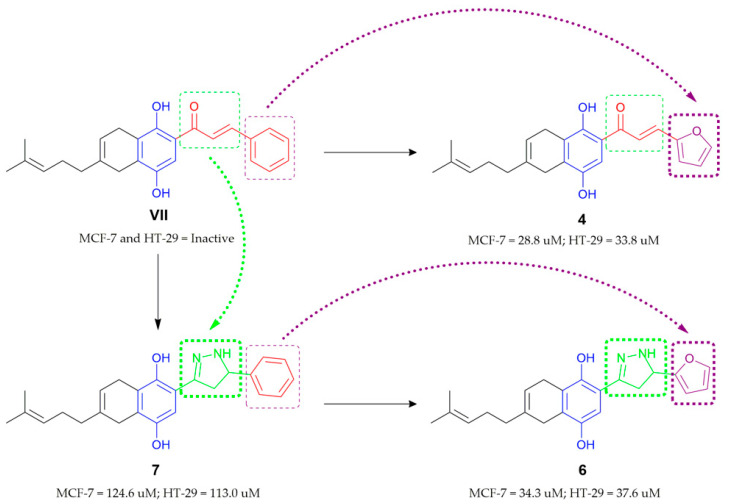
Antiproliferative effects of isosteric replacement and pyrazoline heterocycle incorporation into synthesized chalcones.

PIBHQ derivative **6** generally showed better cytotoxicity values compared to analogous hybrids **7**–**10**, increasing cytotoxic potency against both tumoral cell lines by up to three times (Figure 2). This underscores the importance of the furanyl heterocycle in this series of hybrids. The exception was compound 8, which exhibited a significant pIC_50_ value of 4.51 against the HT-29 cell line. Interestingly, the presence of 2,4-dichloro substituents in hybrid **10** resulted in limited antiproliferative effects, with pIC_50_ values lower than 3.52 against both cancer cell lines, similar to previously reported results [41].

**Figure 3:**
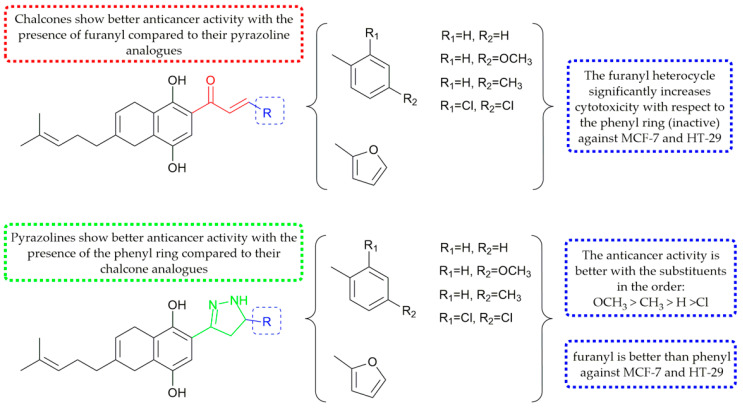
Structure–activity relationship (SAR) of chalcone hybrids and their synthesized pyrazoline derivatives.

Regarding structure–activity relationship (SAR) studies, the isosteric substitution of the benzene ring with the furan heterocycle in the chemical skeletons of chalcone/1,4-benzohydroquinone hybrids was found to be crucial for obtaining cytotoxic molecules. The respective analogues, CBHQs **VII**–**X**, were inactive against both studied cell lines [41]. Additionally, the incorporation of the pyrazoline heterocycle into the chemical structures of CBHQs **VII**–**X** (Figure 3) was significant in producing new cytotoxic molecules, as evidenced by PIBHQs **7**–**9**, which showed pIC_50_ values between 3.90 and 4.51, compared to their inactive precursor chalcones (**VII**–**X**) against MCF-7 and HT-29 cell lines. These findings motivate us to continue developing the designs of new bioactive compounds, leveraging the reactivity of chalcones to incorporate new heterocyclic bioisosteric entities, aiming to obtain new candidates with greater pharmacological potential as anticancer agents.

### 2.3. In Silico Virtual Screening for Potential Antineoplastic Targets of CBHQs and PIBHQs

To obtain information about the potential anticancer mechanisms of the synthesized compounds, we performed molecular docking studies to identify possible biological targets in various cancer-related proteins and calculated their corresponding ∆G*_bin_* values. The searches for the possible binding sites of these cytotoxic derivatives were conducted through virtual screening with proteins involved in antineoplastic processes. Among these targets, we selected proteins overexpressed in breast cancer and colon cancer, including the mesenchymal–epithelial transition factor (c-MET), receptor of the epidermal growth factor (EGFR), fibroblast growth factor receptor 2 (FGFR-2), epidermal growth factor receptor 2 (HER2), tropomyosin receptor kinase A (TRKA), tyrosine protein kinase (TPK), mitogen-activated protein kinases (MAPK1, ERK2, and MEK1), dihydrofolate reductase (DHFR), estrogen receptors (ER), vascular endothelial growth factor receptor 2 (VEGFR-2), and tubulin (TUB), among others [42,43,44,45,46].

The virtual screening performed with the selected proteins (Table S1) revealed that the cytotoxic derivatives present more stable conformations, particularly with protein kinases, with binding energies averaging greater than −8.42 kcal/mol. As shown in Table 2, the derivatives primarily bind strongly to the epidermal growth factor receptor (EGFR), with ∆G*_bin_* values ranging from −11.4 to −8.5 kcal/mol (average, −10.68 kcal/mol). This is followed by protein kinases, with the best bindings to MEK1 having energies between −10.7 and −8.5 kcal/mol (average, −9.95 kcal/mol); CK4, ranging from −10.5 to −9.3 kcal/mol (average, −9.87 kcal/mol); and TPK, ranging from −10.6 to −8.5 kcal/mol (average, −9.72 kcal/mol). Interestingly, PIBHQs **6**–**9** exhibited higher affinity for all the selected protein kinases (Table 2) compared to CBHQ hybrids **4** and **5**, demonstrating the importance of the pyrazoline heterocycle for protein binding. Additionally, in vitro activity studies have shown that pyrazoline derivatives can act as EGFR kinase inhibitors [47,48,49,50].

As detailed in Table 2, the most favorable ∆G*_bin_* values against EGFR and MEK1 were demonstrated by derivative **7** (−11.4 and −10.7 kcal/mol, respectively). The highest ∆G*_bin_* values against CK4, TPK, and TRKA were exhibited by derivative **9** (−10.5, −10.6, and −10.1 kcal/mol, respectively), while compound **6** showed the highest affinity against TPK (−10.3 kcal/mol). Interestingly, PIBHQ hybrids **6**–**9** showed a slight increase in affinity for most protein kinases, with ∆G*_bin_* values exceeding those of CBHQs **4** and **5,** despite their greater cytotoxicity. Most of the cytotoxic hybrids exhibited better binding affinities to the studied proteins, with higher ∆G*_bin_* values compared to the anticancer drugs used as references (anlotinib, crizotinib, and erlotinib), which act as strong kinase inhibitors [51,52,53,54,55,56,57,58,59,60]. Notably, most of the compounds demonstrated superior ΔG*_bin_* values compared to these reference antiproliferative drugs (Table 2).

Despite PIBHQ **10** not exhibiting cytotoxicity against the tested cancer cell lines (Table 1), it demonstrated favorable binding energies within the active sites of kinase proteins, as presented in Table S2. This observation can be attributed to robust hydrogen bond interactions between the free hydroxyl groups of the hydroquinone system and oxygen- or nitrogen-containing groups within the proteins, as well as van der Waals interactions between the chloride of the phenyl group and residues, as illustrated in Figures S25–S27.

### 2.4. Binding Sites and Docking of Synthesized Cytotoxic Hybrids in EGFR, MEK1, CK4, and TPK Targets

A more detailed analysis comparing the binding energies of PIBHQs with respect to their CBHQ precursors against EGFR, MEK1, CK4, and TPK (Table S2) indicates that most compounds show an increase in affinities against protein kinases, with ∆G*_bin_* average values of less than -8.50 kcal/mol. The most favorable ∆G*_bin_* values were shown against EGFR and MEK1, with average ∆G*_bin_* energies of −10.68 and −9.95 kcal/mol, respectively, followed by c-MET and TRKA (−9.82 and −9.72 kcal/mol, respectively). These findings suggest that these hybrids might serve as potent inhibitors of EGFR, MEK1, and c-MET, all of which are overexpressed in certain types of cancer, including human breast and colorectal cancer [42,61,62,63]. Furthermore, PIBHQs **6**–**9** showed higher affinity for all the selected protein kinases (Table 2) compared to CBHQ hybrids **4** and **5.** These findings reaffirm that the presence of the pyrazoline cycle in hybrids **7**–**9** is crucial for increased affinity to the selected protein kinases compared to the precursor chalcones (**VII**–**IX**).

**Figure 4:**
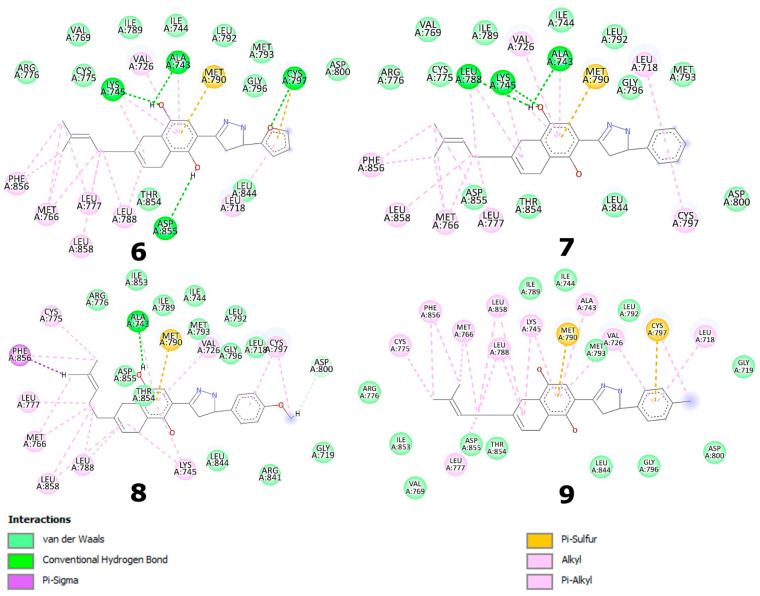
Two-dimensional maps of H bonds and hydrophobic interactions of CNQ **6**, **7**, **8**, and **9** with EGFR residues. Van der Waals, Pi–anion, Pi–sigma, Pi–Pi stacked, and Pi–alkyl interactions are considered hydrophobic.

Detailed configurations of the binding sites, along with the amino acids involved in the docking of synthesized cytotoxic hybrids and their corresponding ΔG*_bin_* values for EGFR, MEK1, and c-MET, are presented in Table 3 and depicted in 2D maps in Figure 4. Additionally, 3D docking complexes of c-MET with **6**–**9** are illustrated in Figure 4. Complementary 2D maps for complexes involving **4** and **5** can be found in Figure S17, and binding site interactions of the synthesized cytotoxic hybrids with amino acids of TPK, TRKA, and CK4 are outlined in Table S2.

Overall, as demonstrated in Table 2 and Table 3, PBHQ derivative **7** exhibited superior binding affinity for biological targets, with the best average ΔG*_bin_* values for EGFR and MEK1, due to the presence of the C1 and C4 hydroxyl groups of the hydroquinone ring. These groups interacted with amino acid residues through hydrogen bonding. For instance, the hydroxyl groups of the hydroquinone ring in derivatives **7** and **8** interacted with Ala743 of EGFR (Figure 4). Specifically, PBHQs **6**, **7**, **8**, and **9** displayed excellent binding affinities for c-MET, with ΔG*_bin_* values of −11.3, −11.4, −11.0, and −11.2 kcal/mol, respectively. Peptide sequences surrounding the PIBHQs revealed consistent docking in the same region of the enzyme, defined by residues Ala743 and Met790. All the compounds, including **4** and **5**, engaged in hydrogen bonding with EGFR residues, as well as various other interactions, including van der Waals, Pi–anion, Pi–sigma, Pi–Pi stacked, and Pi–alkyl interactions.

**Figure 5:**
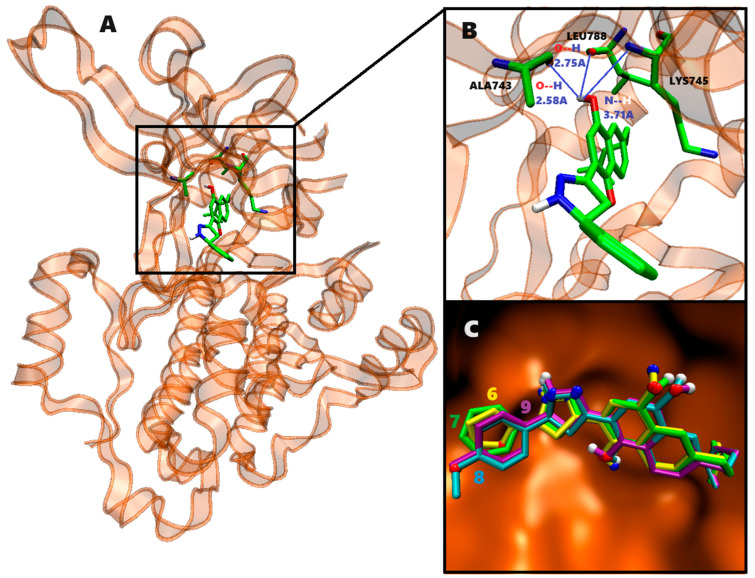
(**A**) Visualization of the potential binding site of PBHQ hybrid **7** into EGFR; (**B**) detail of its H bonding with Ala743, Lys745, and Leu788; (**C**) superimposition of the docking poses for PHBQ hybrids 6 (yellow), 7 (green), 8 (cyan), and 9 (purple).

According to hydrogen bonds, residues Ala743, Lys745, and Leu788 were most commonly involved in interactions with EGFR, serving as hydrogen bond donors toward hydroxyl groups from the hydroquinone moieties of **4**, **6**, **7**, and **8**; the pyrazoline moiety of **8**; or the acetyl group of **5** (Figure 5 and Figure S25). In the case of MEK1, residues Val211 and Ser212 interacted with hydroxyl groups from the hydroquinone of **4** and **7**, respectively, while Lys97 interacted with the nitrogen from the pyrazoline moieties of **8** and **9** through hydrogen bonds (Figure S26). c-MET exhibited interactions with residues such as Arg1227, which primarily engaged in hydrogen bonds with hydroxyl groups from the hydroquinones of **4** and **9**, as well as Asn1167, which interacted with the furan groups of **4** and **5**, and Asn1167 and Arg1227 with the acetyl group of **5** (Figure S27). Tyr1234 and Arg1166 interacted with the oxygen of the methoxy group of **8**.

Aromatic interactions, similar to hydrogen bonds, play a crucial role in ligand–protein interfaces. Many contemporary ligand docking programs implicitly account for aromatic stacking through van der Waals and Coulombic potentials [41,64]. Residues Met766, Met790, and Cys797 of EGFR were notably involved in these interactions, engaging in π–sulfur interactions with the aromatic rings of hydroquinone systems in **4**–**9 b** (Figure 4 and Figure S25). Additionally, the sulfur of Met790 interacts with the rings of the hydroquinone in **4** and **5**, in addition to Cys797 and Met766 interacting with the furan group in **4** and **5**, respectively, through π–sulfur interactions (Figure S25).

In the case of MEK1, Phe209 was the primary residue involved in aromatic interactions, participating in π–π stacking with the furan group in **5** and the aromatic ring of the phenyl group in **9** and **10.** The sulfur of Met143 interacted with the furan group in **5** and the aromatic ring of the phenyl group in **8** and **10** through π–sulfur interactions. Leu215 and Met219 interacted through π–alkyl interactions with the aromatic rings of the quinone moiety in **5–9**, while Asp208 interacted through π–anion interactions with the aromatic ring of the pyrazoline heterocycle in **9** and **10** (Figure S26). Lastly, Phe1089 and Phe1223 played a prominent role in c-MET interactions, engaging in π–π stacking with the aromatic ring of the pyrazoline heterocycle in **6**, **9**, and **10**. Arg1227 interacted through π–alkyl interactions with the aromatic rings of the quinone moiety in **4** and **5**, while Val1092 interacted through π–sigma interactions with the aromatic rings of the pyrazoline heterocycle in **7** and **9**. Asp1164 interacted through π–anion interactions with the hydroquinone ring in **9** and **10** (Figure S27).

To validate the binding sites of the synthesized cytotoxic hybrids within the kinases, we conducted a comparative analysis of PIBHQ **7** complexes with those of known kinase ligands. The results revealed that the binding regions of PIBHQ **7**, indeed, overlap with the catalytic sites of the target enzymes, sharing a common set of contacts with the respective inhibitors (Table 4). Notably, the active site residues involved in these interactions include Leu718, Val726, Ala743, Lys745, Met766, Cys775, Arg776, Leu777, Leu788, Met790, Met793, Gly796, Leu844, Thr854, Asp855, Phe856, and Leu858 for EGFR; Lys97, Leu115, Leu118, Val127, Phe129, Ile141, Met143, Asp190, Cys207, Asp208, Phe209, Gly210, Val211, Ser212, Leu215, Ile216, and Met219 for MEK1; and Phe1089, Val1092, Lys1110, Leu1157, Met1211, Phe1223, and Ala1226 for c-MET. These residues served as common contact points for PIBHQ **7** and ligands 1, 2, and 3 in all three enzymes.

**Figure 6:**
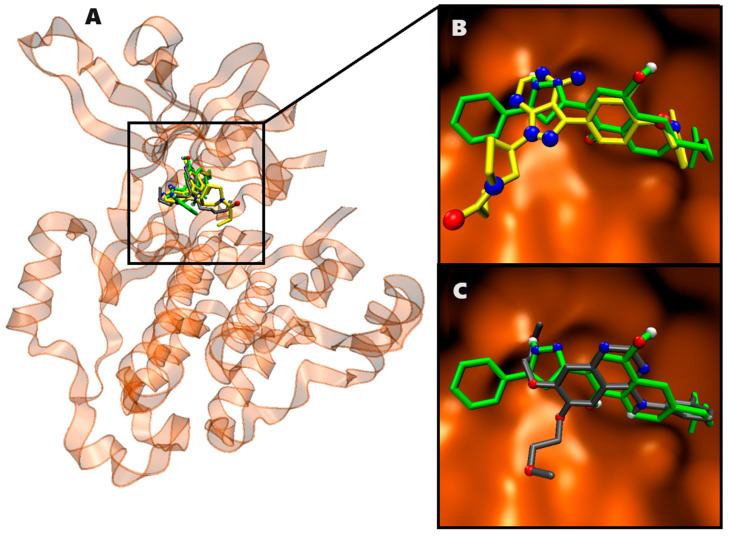
(**A**): Overlapping of the docking poses for PIBHQ hybrid **7** (green), ligand 1 (yellow), and erlotinib (grey) into c-MET. Superimposition of the docking poses for (**B**) **6c** and ligand 1, as well as (**C**) **6c** and erlotinib.

We compared the binding site and affinity of PIBHQ **7** with anticancer drugs used as inhibitors of EGFR, MEK1, and c-MET [54,55,57]. Notably, the energetic aspects of these interactions favored PIBHQ **7** in comparison to erlotinib, with a favorable energy difference of 1.5 kcal/mol for c-MET and 2.8 kcal/mol for EGFR. Moreover, **7** exhibited better in silico affinity than anlotinib for MEK1 and crizotinib for MEK1, with favorable energy differences of 1.0 kcal/mol and 0.3 kcal/mol, respectively. Importantly, the aromatic ring within the pyrazoline moiety, as well as the hydroxyl groups from the hydroquinone moiety of PIBHQs, plays a pivotal role in these interactions, directly contributing to the overlap with the ligands at the catalytic sites of the enzymes (Figure 6). This crucial involvement of the pyrazoline moiety is consistently observed in the case of EGFR, as well as MEK1 and c-MET (Figures S26 and S27).

Furthermore, it is possible to speculate that CBHQ **5** may act as a prodrug. It could undergo hydrolysis through deacetylation within the cell, catalyzed by a “deacetylase” enzyme, releasing the molecule in the form of CBHQ **4**. Subsequently, these benzohydroquinone compounds might exhibit an inhibitory effect on cancer-related kinases. This assumption is supported by their favorable binding energies in the active site of the kinase domain of EGFR, as detailed in Figure S25 and Table 4.

### 2.5. In Silico Drug Likeness, Toxicity Risks, and ADME Predictions

Drug-likeness scores for compounds **4**–**9** were computed using the DataWarrior algorithm, and the results are presented in Table 5. Notably, derivatives **7** and **8** stand out as the only compounds with positive drug-likeness values of 0.53 and 0.83, respectively. This significant finding suggests that compounds **7** and **8** could be promising lead candidates for further investigation. It is noteworthy that compounds **7** and **8** incorporate essential structural elements, such as the hydroquinone and pyrazoline fragments, which are commonly found in approved drugs. Additionally, compound **8** features a phenyl fragment with a methoxy group at position 3. These substituents are known to contribute significantly to the enhancement of the antineoplastic cytotoxicity of potential anticancer agents.

In terms of toxicity risks, CBHQs **4** and **5** are likely to exhibit a high level of mutagenic risk, whereas PIBHQs **6**–**9** are expected to have no adverse effects (Table 5). The high irritant risk associated with compound **5** can be attributed to the acetylation in the hydroquinone moiety.

The predicted values for several pharmacokinetic parameters of compounds **4**–**9** related to oral absorption, Caco-2 cell permeability, blood–brain barrier permeability, and binding to human serum albumin, among others, are summarized in Table S3. These ADME descriptor values indicate that the percentage of predicted oral absorption for these compounds ranges from 94% to 100%, suggesting good oral bioavailability. Furthermore, all the evaluated compounds demonstrate good to excellent predicted values for Caco-2 cell permeability, with QPlogBB values falling between −2.6 and −0.68. Additionally, all the tested compounds are within the range of interaction with human serum albumin, suggesting their potential transport by plasma proteins to the target site.

However, it is worth noting that all compounds may block HERG K+-channels, which play a crucial role in cardiac repolarization, potentially increasing the risk of cardiac arrhythmias. Moreover, compound **5** is expected to have sufficient solubility in water, while compounds **4**–**9** are considered higher-lipophilicity compounds, enhancing their ability to penetrate cell membranes. In terms of compliance with Jorgensen’s rule of three, most compounds remain within permissible limits, except for PIBHQs **7**, **9**, and **10**, which exhibit three violations.

Moreover, nearly all the evaluated compounds meet Lipinski’s rule of five and its Weber extension criteria, except for compounds **4** and **7**–**9** (QPlog/Po/w > 5). However, even these compounds have violations that fall within acceptable limits (Table S4). These results collectively suggest that, from a pharmacokinetic perspective, most of these compounds hold promise as potential candidates for preclinical assays.

## 3. Materials and Methods

### 3.1. Chemistry

All chemical reactions were carried out from the starting substrate, compound 1 [65], following the synthesis route to obtain chalcones from precursor 2, as previously described [41]. The reaction progress was monitored by thin-layer chromatography with 60 F254 silica gel plates (Merck, TLC silica gel 60 F254). Column chromatography (CC) purification was performed with silica gel 60 (230–400 mesh, Merck). Melting points were determined on a Stuart SMP 10 apparatus (Stone, Staffordshire, UK) and were uncorrected. IR spectra were recorded on a Perkin Elmer FT IR 1600 spectrophotometer (Perkin Elmer, Norwalk, CN, USA) as a film generated by applying a solution of the derivate in CH_2_Cl_2_ on NaCl disks, then by evaporation of the solvent under an IR lamp. ^1^H NMR spectra were recorded operating at 400.13 MHz, while ^13^C NMR spectra were recorded at 100.62 MHz on a Bruker Avance 400 Digital NMR spectrometer (Bruker/Analytic, Karlsruhe, Germany) in CDCl_3_ using TMS as an internal reference. Chemical shift (δ) values are specified as parts per million (ppm), followed by multiplicity and coupling constant (*J*) in Hertz (Hz). Elemental analyses of C, H, and N were determined by a Perkin Elmer 2400 Series II CHN Elemental Analyzer (Perkin Elmer Inc., Waltham, MA02451, 21,929 USA).

#### 3.1.1. Procedure for the Synthesis and Characterization of Compound **3**

##### Synthesis of (*E*)-3-(Furan-2-yl)-1-{1-hydroxy-6-(4-methylpent-3-en-1-yl)-4-[(tetrahydro-2H-pyran-2-yl)oxy]-5,8-dihydronaphthalen-2-yl}prop-2-en-1-one (**3**)

A solution containing precursor 2 (1.00 mmol) and barium hydroxide octahydrate (1.00 mmol) in ethanol (8 mL) was stirred for 10 min. Then, 2-furaldehyde (1.15 mmol) was added, and the mixture was stirred for 30 min at 90 °C. After the reaction, the mixture was added to an ice/water bath, vacuum-filtered, and purified by recrystallization using methanol as the solvent, obtaining pure product 3 as an orange solid (283 mg, 63%): m.p.116–117 °C. IR υ_max_ cm^−1^ (film) 3374 (O-H), 1639 (C=O), 1583 (C=C). ^1^H NMR (CDCl_3_, TMS, ppm) δ 1.69 (m, 10H, 2CH_3_, 2CH_2_, H13, H14, H3′, H4′), 1.94 (m, 2H, CH_2_, H2′), 2.19 (m, 4H, 2CH_2_, H10, H9), 3.34 (m, 4H, 2CH_2_, H8, H5), 3.70 (m, 1H, CH_2_, H5′), 3.95 (m, 1H, CH_2_, H5′), 5.19 (t, 1H, *J* = 6.8 Hz, CH, H11), 5.43 (t, 1H, *J* = 3.3 Hz, CH, H1′), 5.67 (s, 1H, CH, H7), 6.54 (dd, 1H, *J* = 3.4 Hz, *J* = 1.8 Hz, CH, H20), 6.75 (d, 1H, *J* = 3.4 Hz, CH, H19), 7.50 (d, 2H, *J* = 15.2 Hz, 2CH, H3, H16), 7.57 (d, 1H, *J* = 1.5 Hz, CH, H21), 7.68 (d, 1H, *J* = 15.2 Hz, CH, H17), 13.18 (s, 1H, OH, H1). ^13^C NMR (CDCl_3_, TMS, ppm) δ 17.7 (C13), 19.0 (C3′), 24.8 (C8), 25.3 (C4′), 25.7 (C14), 26.2 (C10), 28.4 (C5), 30.7 (C2′), 37.2 (C9), 62.0 (C5′), 97.1 (C1′), 110.7 (C3), 112.8 (C20), 116.3 (C2), 116.6 (C19), 117.7 (C11), 118.2 (C16), 124.1 (C7), 125.0 (C8a), 130.6 (C17), 131.8 (C4a), 133.5 (C12), 135.3 (C6), 145.2 (C21), 146.3 (C4), 151.7 (C18), 156.9 (C1), 192.8 (C15). Elemental analysis calculated for C_28_H_32_O_5_: C, 74.97; H, 7.19. Found: C, 74.99; H, 7.16.

#### 3.1.2. Procedure for the Synthesis and Characterization of Compound **4**

##### Synthesis of (*E*)-1-[1,4-Dihydroxy-6-(4-methylpent-3-en-1-yl)-5,8-dihydronaphthalen-2-yl]-3-(furan-2-yl) prop-2-en-1-one (**4**)

Acid monohydrate 4-toluenesulfonic (3.00 mmol) was added to a solution of compound 3 (1.00 mmol) in methanol (12 mL). The reaction mixture was stirred for 3 h at room temperature. After the reaction, the mixture was added to an ice/water bath, vacuum-filtered, and purified by recrystallization using methanol as the solvent, obtaining a red solid (335 mg, 92%): m.p.124–125 °C. IR υ_max_ cm^−1^ (film) 3381 (O-H), 1634 (C=O), 1581 (C=C). ^1^H NMR (CDCl_3_, TMS, ppm) δ 1.68 (m, 6H, 2CH_3_, H13, H14), 2.17 (m, 4H, 2CH_2_, H10, H9), 3.32 (m, 4H, 2CH_2_, H8, H5), 4.80 (s, 1H, OH, H4), 5.17 (t, 1H, *J* = 6.7 Hz, CH, H11), 5.68 (s, 1H, CH, H7), 6.54 (s, 1H, CH, H20), 6.74 (d, 1H, *J* = 3.5 Hz, CH, H19), 7.18 (s, 1H, CH, H3), 7.45 (d, 1H, *J* = 15.1 Hz, CH, H16), 7.54 (s, 1H, CH, H21), 7.65 (d, 1H, *J* = 15.1 Hz, CH, H17), 13.00 (s, 1H, OH, H1). ^13^C NMR (CDCl_3_, TMS, ppm) δ 17.8 (C13), 24.8 (C8), 25.7 (C14), 26.1 (C10), 28.2 (C5), 37.2 (C9), 110.5 (C3), 112.8 (C20), 116.4 (C2), 116.8 (C19), 117.9 (C11, C16), 124.0 (C7), 125.2 (C8a), 130.6 (C17), 131.9 (C12), 132.7 (C4a), 132.9 (C6), 144.8 (C4), 145.3 (C21), 151.6 (C18), 156.0 (C1), 192.5 (C15). Elemental analysis calculated for C_23_H_24_O_4_: C, 75.80; H, 6.64. Found: C, 75.82; H, 6.65.

#### 3.1.3. Procedure for the Synthesis and Characterization of Compound **5**

##### Synthesis of (*E*)-2-[3-(Furan-2-yl) prop-2-enoyl]-6-(4-methylpent-3-en-1-yl)-5,8-dihydronaphthalene-1,4-diyl diethanoate (**5**)

Acetic anhydride (1.00 mL, 10.6 mmol) was added to a solution of compound 4 (0.50 mmol) in pyridine (1.25 mL), and the reaction mixture was maintained in the dark with occasional stirring for 16 h at room temperature. After the reaction, the mixture was added to an ice/water bath, dissolved with CH_2_Cl_2_ (30 mL), and subjected to successive extractions with 10% HCl solution (2 × 15 mL) and H_2_O (2 × 10 mL) until neutral pH of the aqueous phase was achieved. The organic phase was dried with anhydrous Na_2_SO_4_, filtered, and evaporated under reduced pressure. The crude product was purified by column chromatography with hexane/ethyl acetate 2:1 as eluent, obtaining an orange solid (215 mg, 96%): m.p.120–121 °C. IR υ_max_ cm^−1^ (film) 1766 (C=O), 1604 (C=C). ^1^H NMR (CDCl_3_, TMS, ppm) δ 1.67 (m, 6H, 2CH_3_, H13, H14), 2.26 (m, 10H, 2CH_3_, 2CH_2_, H’2, H’4, H10, H9), 3.22 (m, 4H, 2CH_2_, H5, H8), 5.14 (s, 1H, CH, H11), 5.59 (s, 1H, CH, H7), 6.51 (s, 1H, CH, H20), 6.71 (d, 1H, *J* = 3.6 Hz, CH, H19), 7.10 (d, 1H, *J* = 15.5 Hz, CH, H16), 7.35 (s, 1H, CH, H3), 7.43 (d, 1H, *J* = 15.5 Hz, CH, H17), 7.53 (s, 1H, CH, H21). ^13^C NMR (CDCl_3_, TMS, ppm) δ 17.8 (C13), 20.8 (C2′), 20.9 (C4′), 25.3 (C8), 25.7 (C14), 26.0 (C10), 27.9 (C5), 36.9 (C9), 112.7 (C20), 116.6 (C16), 116.9 (C19), 120.7 (C3), 122.1 (C11), 123.8 (C7), 129.9 (C4a), 130.5 (C2), 131.4 (C17), 132.0 (C12), 133.1 (C8a), 133.3 (C6), 144.5 (C4), 145.2 (C21), 145.7 (C1), 151.3 (C18), 169.0 (C3′), 169.1 (C1′), 189.5 (C15). Elemental analysis calculated for C_27_H_28_O_6_: C, 72.30; H, 6.29. Found: C, 72.32; H, 6.27.

#### 3.1.4. General Procedure for the Synthesis and Characterization of Compounds **6**, **7**, **8**, **9**, and **10**

Hydrazine monohydrate (1.50 mmol) was added to a solution of the respective compounds (**4**, **VII**, **VIII**, **IX**, and **X**) (0.50 mmol) in ethanol (10 mL). The reaction mixture was refluxed for 5 h. After the reaction time, the mixture was cooled in an ice/water bath. The resulting solid was extracted with ethyl acetate (2 × 20 mL). After separating the phases, the organic phase was dried with anhydrous Na_2_SO_4_, filtered, and evaporated under reduced pressure, yielding the respective impure products of 6, 7, 8, 9, and 10.

##### Synthesis of 2-[5-(Furan-2-yl)-4,5-dihydro-1*H*-pyrazol-3-yl]-6-(4-methylpent-3-en-1-yl)-5,8-dihydronaphthalene-1,4-diol (**6**)

This compound was synthesized by a general procedure using precursor 4 and was purified by recrystallization using ethanol as a solvent, obtaining a yellow solid (136 mg, 71%): m.p.115–116 °C. IR υ_max_ cm^−1^ (film) 3328 (O-H), 3328 (N-H), 1634 (C=N), 1599 (C=C). ^1^H NMR (CDCl_3_, TMS, ppm) δ 1.67 (m, 6H, 2CH_3_, H13, H14), 2.16 (m, 4H, 2CH_2_, H9, H10), 3.29 (m, 6H, 3CH_2_, H8, H5, H16), 4.88 (t, 1H, *J* = 8.9 Hz, CH, H17), 5.17 (s, 1H, CH, H11), 5.67 (s, 1H, CH, H7), 6.23 (d, 1H, *J* = 3.3 Hz, CH, H19), 6.32 (s, 1H, CH, H20), 6.52 (s, 1H, CH, H3), 7.37 (s, 1H, CH, H21), 10.77 (s, 1H, OH, H1). ^13^C NMR (CDCl_3_, TMS, ppm) δ 17.8 (C13), 25.2 (C8), 25.7 (C14), 26.2 (C10), 27.6 (C5), 37.3 (C9), 37.9 (C16), 55.5 (C17), 106.4 (C19), 110.3 (C20), 110.4 (C3), 112.5 (C2), 118.1 (C11), 123.5 (C8a), 124.1 (C7), 125.1 (C4a), 131.8 (C12), 133.2 (C6), 142.5 (C21), 145.3 (C4), 149.2 (C1), 154.0 (C18), 155.1 (C15). Elemental analysis calculated for C_23_H_26_N_2_O_3_: C, 72.99; H, 6.92; N, 7.40. Found: C, 72.97; H, 6.95; N, 7.43.

##### Synthesis of 6-(4-Methylpent-3-en-1-yl)-2-(5-phenyl-4,5-dihydro-1*H*-pyrazol-3-yl)-5,8-dihydronaphthalene-1,4-diol (**7**)

This compound was synthesized by a general procedure using precursor VII and was purified by recrystallization using ethanol as a solvent, obtaining a brown solid (120 mg, 63%): m.p.136–137 °C. IR υ_max_ cm^−1^ (film) 3341 (O-H), 1627 (C=N), 1600 (C=C). ^1^H NMR (CDCl_3_, TMS, ppm) δ 1.69 (m, 6H, 2CH_3_, H13, H14), 2.19 (m, 4H, 2CH_2_, H10, H9), 3.06 (dd, 1H, *J*_AB_ = 16.4 Hz, *J*_AX_ = 8.4 Hz, CH_2_, H16_A_), 3.37 (m, 5H, 3CH_2_, H8, H5, H16_B_), 4.85 (dd, 1H, *J*_BX_ = 10.7 Hz, *J*_AX_ = 8.4 Hz, CH, H17_X_), 5.19 (s, 1H, CH, H11), 5.69 (s, 1H, CH, H7), 6.50 (s, 1H, CH, H3), 7.35 (s, 6H, 5CH, OH, H19, H20, H21, H22, H23, H4), 10.84 (s, 1H, OH, H1). ^13^C NMR (CDCl_3_, TMS, ppm) δ 17.2 (C13), 25.2 (C8), 25.7 (C14), 26.2 (C10), 29.7 (C5), 37.3 (C9), 48.6 (C16), 62.6 (C17), 110.2 (C3), 112.2 (C2), 118.2 (C11), 124.1 (C7), 126.3 (C21), 126.9 (C8a), 128.0 (C19, C23), 128.8 (C4a), 129.0 (C20, C22), 131.7 (C12), 133.1 (C6), 142.2 (C18), 145.2 (C4), 149.2 (C1), 154.3 (C15). Elemental analysis calculated for C_25_H_28_N_2_O_2_: C, 77.29; H, 7.26; N, 7.21. Found: C, 77.33; H, 7.28; N, 7.18.

##### Synthesis of 2-[5-(4-Methoxyphenyl)-4,5-dihydro-1*H*-pyrazol-3-yl]-6-(4-methylpent-3-en-1-yl)-5,8-dihydronaphthalene-1,4-diol (**8**)

This compound was synthesized by a general procedure using precursor VIII and was purified by recrystallization using ethanol as a solvent, obtaining a brown solid (130 mg, 62%): m.p.128–129 °C. IR υ_max_ cm^−1^ (film) 3376 (O-H), 1667 (C=N), 1612 (C=C). ^1^H NMR (CDCl_3_, TMS, ppm) δ 1.67 (m, 6H, 2CH_3_, H13, H14), 2.18 (m, 4H, 2CH_2_, H10, H9), 3.02 (dd, 1H, *J*_AB_ = 16.5 Hz, *J*_AX_ = 8.2 Hz, CH_2_, H16_A_), 3.21 (m, 2H, CH_2_, H5), 3.39 (m, 3H, CH_2_, H8, H16_B_), 3.81 (s, 3H, CH_3_, H24), 4.79 (dd, 1H, *J*_BX_ = 10.2 Hz, *J*_AX_ = 8.2 Hz, CH, H17_X_), 5.16 (s, 1H, CH, H11), 5.68 (s, 1H, CH, H7), 6.50 (s, 1H, CH, H3), 6.88 (d, 2H, *J* = 8.9 Hz, 2CH, H20, H22), 7.26 (d, 3H, *J* = 8.9 Hz, 2CH, OH, H19, H23, H4), 10.78 (s, 1H, OH, H1). ^13^C NMR (CDCl_3_, TMS, ppm) δ 17.7 (C13), 25.2 (C8), 25.7 (C14), 26.2 (C10), 27.6 (C5), 37.3 (C9), 41.5 (C16), 55.3 (C24), 62.2 (C17), 110.2 (C3), 112.8 (C2), 114.3 (C20, C22), 118.2 (C11), 123.4 (C8a), 124.1 (C7), 124.7 (C18), 127.5 (C19, C23), 131.8 (C4a), 133.1 (C12), 134.2 (C6), 145.2 (C4), 149.2 (C1), 154.6 (C15), 159.4 (C21). Elemental analysis calculated for C_26_H_30_N_2_O_3_: C, 74.61; H, 7.22; N, 6.69. Found: C, 74.65; H, 7.23; N, 6.67.

##### Synthesis of 6-(4-Methylpent-3-en-1-yl)-2-[5-(4-methylphenyl)-4,5-dihydro-1*H*-pyrazol-3-yl]-5,8-dihydronaphthalene-1,4-diol (**9**)

This compound was synthesized by a general procedure using precursor IX and was purified by recrystallization using ethanol as a solvent, obtaining a brown solid (137 mg, 67%): m.p.119–120 °C. IR υ_max_ cm^−1^ (film) 3340 (O-H), 1634 (C=N), 1600 (C=C). ^1^H NMR (CDCl_3_, TMS, ppm) δ 1.68 (m, 6H, 2CH_3_, H13, H14), 2.19 (m, 4H, 2CH_2_, H10, H9), 2.36 (s, 3H, CH_3_, H24), 3.05 (dd, 1H, *J*_AB_ = 16.3 Hz, *J*_AX_ = 8.1 Hz, CH_2_, H16_A_), 3.36 (m, 5H, 3CH_2_, H5, H8, H16_B_), 4.81 (t, 1H, *J* = 8.8 Hz, CH, H17_X_), 5.17 (t, 1H, *J* = 6.2 Hz, CH, H11), 5.69 (s, 1H, CH, H7), 6.50 (s, 1H, CH, H3), 7.20 (m, 5H, 4CH, OH, H19, H20, H22, H23, H4), 10.85 (s, 1H, OH, H1). ^13^C NMR (CDCl_3_, TMS, ppm) δ 17.8 (C13), 21.1 (C24), 25.2 (C8), 25.7 (C14), 26.2 (C10), 27.6 (C5), 37.3 (C9), 41.6 (C16), 62.4 (C17), 110.2 (C3), 112.8 (C2), 118.2 (C11), 123.4 (C8a), 124.1 (C7), 124.7 (C12), 126.2 (C19, C23), 129.6 (C20, C22), 131.8 (C4a), 133.1 (C18), 137.8 (C6), 139.9 (C21), 145.2 (C4), 149.2 (C1), 154.5 (C15). Elemental analysis calculated for C_26_H_30_N_2_O_2_: C, 77.58; H, 7.51; N, 6.96. Found: C, 77.60; H, 7.47; N, 6.98.

##### Synthesis of 2-[5-(2,4-Dichlorophenyl)-4,5-dihydro-1*H*-pyrazol-3-yl]-6-(4-methylpent-3-en-1-yl)-5,8-dihydronaphthalene-1,4-diol (**10**)

This compound was synthesized by a general procedure using precursor X and was purified by recrystallization using methanol as a solvent, obtaining a brown solid (174 mg, 75%): m.p.138–139 °C. IR υ_max_ cm^−1^ (film) 3349 (O-H), 1627 (C=N), 1600 (C=C). ^1^H NMR (CDCl_3_, TMS, ppm) δ 1.67 (m, 6H, 2CH_3_, H13, H14), 2.16 (m, 4H, 2CH_2_, H10, H9), 2.84 (dd, 1H, *J*_AB_ = 16.7 Hz, *J*_AX_ = 9.4 Hz, CH_2_, H16_A_), 3.27 (m, 4H, 2CH_2_, H5, H8), 3.61 (dd, 1H, *J*_AB_ = 16.7 Hz, *J*_BX_ = 10.7 Hz, CH_2_, H16_B_), 5.18 (m, 2H, 2CH, H11, H17_X_), 5.66 (s, 1H, CH, H7), 6.48 (s, 1H, CH, H3), 7.24 (dd, 1H, *J* = 8.37 Hz, *J* = 1.95 Hz, CH, H22), 7.41 (d, 1H, *J* = 1.94 Hz, CH_2_, H20), 7.53 (d, 1H, *J* = 8.77 Hz, CH, H23), 7.66 (s, 1H, OH, H4), 10.76 (s, 1H, OH, H1). ^13^C NMR (CDCl_3_, TMS, ppm) δ 17.8 (C13), 25.1 (C8), 25.7 (C14), 26.2 (C10), 27.6 (C5), 37.2 (C9), 40.2 (C16), 58.9 (C17), 110.2 (C3), 112.4 (C2), 118.1 (C11), 123.4 (C8a), 124.1 (C7), 125.1 (C4a), 127.7 (C22), 128.2 (C20), 129.4 (C23), 131.8 (C12), 133.2 (C21), 133.4 (C19), 134.0 (C18), 137.9 (C6), 145.3 (C4), 149.1 (C1), 154.6 (C15). Elemental analysis calculated for C_25_H_26_Cl_2_N_2_O_2_: C, 65.65; H, 5.73; N, 6.12. Found: C, 65.68; H, 5.78; N, 6.17.

### 3.2. Antiproliferative Assay

Human cell lines HT-29 (human colon adenocarcinoma, ATCC HTB-38) and MCF-7 (human breast adenocarcinoma, ATCC HTB-22) were obtained from the American Type Culture Collection (Manassas, VA, USA) and were maintained in Dulbecco’s Modified Eagles Medium (DMEM) and supplemented with 10% FBS, 100 U/mL penicillin, and 100 μg/mL streptomycin. Cells were subcultured, and antiproliferative assays were carried out following the experimental conditions we previously described [38]. The absorbance at 490 nm was recorded using a Varioskan Flash Multimode Reader (Thermo Scientific, Waltham, MA, USA). To obtain IC_50_ values for each compound, dose–response curves were constructed for both cell lines (MCF-7 and HT-29). Results are reported as the mean value of three independent experiments, with each variation performed in triplicate. Doxorubicin was included as a reference anticancer compound in all evaluations.

### 3.3. Computational Details

#### 3.3.1. Ligand Preparation

The 3D structure of each compound was prepared using ChemDraw Ultra version 12.0, as previously described [38]. The hydrophobicity index (cLogP), drug-likeness values, and toxicity risks were predicted using DataWarrior algorithms [66,67].

#### 3.3.2. In Silico ADME Prediction

Pharmacokinetic parameters were calculated using QikProp (QP) version 4.3 of the Schrodinger suite, based on Lipinski’s rule of five and its extensions, as previously described [38].

#### 3.3.3. Macromolecule Selection and Retrieval

The crystal structures of 16 selected proteins (Table S1), including growth factor receptors, transcription regulators, and enzymes (such as reductases, oxidases, and kinases) were retrieved from the Protein Data Bank [68]. These proteins are overexpressed in some malignancies, including breast and colon adenocarcinoma, as described in the literature [43,44,45,46,61,69,70,71,72,73,74,75,76,77].

#### 3.3.4. Molecular Docking of Ligand–Protein Interactions

Virtual screening was performed using Autodock Vina, a target-specific scoring method useful for virtual screening [78]. All chalcone–pyrazoline–hydroquinone hybrids were docked into a set of proteins with known 3D structures to identify potential inhibitors. Both ligands and proteins were prepared using AutoDock Tools version 1.5.7 (ADT), as previously described [38,79,80]. The binding sites and energies of each compound were predicted for each receptor using Autodock Vina [78]. Graphic analysis of the molecular coupling studies was performed using Visual Molecular Dynamics 1.9 (VMD) [80] and Discovery Studio Biovia 2021 [81].

## 4. Conclusions

This study presents a novel series of chalcone-1,4-benzohydroquinones (CBHQs) synthesized by replacing the aryl group of the β-carbon of the chalcone with the furan heterocycle. The synthetic process involved the classic Claisen–Schmidt condensation reaction. Additionally, the α, β-unsaturated carbonyl system of the CBHQs underwent a conjugated addition with hydrazine to yield pyrazoline-1,4-benzohydroquinone hybrids (PIBHQs). CBHQs **4** and **5** displayed superior pIC_50_ values in MCF-7 and HT-29 cells compared to PIBHQs **6**–**9**, except for CBHQ **6**; however, compound **8** exhibited higher cytotoxic activity against HT-29 cells. This indicates that the chalcone system and the acetylation of aromatic rings enhance the antiproliferative activity of the newly synthesized hybrid derivatives against both the MCF-7 and HT-29 cancer cell lines. Moreover, the substitution of the benzene ring by the furan heterocycle in the chemical skeletons of CBHQ hybrids was crucial for obtaining antineoplastic compounds, as their respective analogues, **VII**–**X**, showed inactivity against both cell lines.

From a theoretical standpoint, the binding energy of cancer-related proteins with CBHQs and PIBHQs was found to be generally higher for kinases such as EGFR, MEK1, and c-MET, with ΔG*_bin_* values ranging from −11.4 to −8.5 kcal/mol. In this regard, the synthesized hybrids could be promising chemotherapeutic agents, potentially targeting kinase pathways. Furthermore, they could serve as scaffolds for the development of novel multi-target anticancer agents. The favorable predictions of physicochemical and pharmacokinetic parameters for most of these compounds, aligning well with previous in vitro antiproliferative results, underscore their potential as promising candidates for preclinical assays.

## Figures and Tables

**Table 1:** In vitro cytotoxicity data for compounds **4**–**10** in MCF-7 breast adenocarcinoma and HT-29 colon adenocarcinoma cells.

Compounds	MCF-7		HT-29	
	IC_50_, μM ^[a]^	pIC_50_ ^[b]^	IC_50_, μM	pIC_50_
**4**	**28.8 ± 0.84**	**4.54**	**33.8 ± 0.94**	**4.47**
**5**	**21.8 ± 0.41**	**4.66**	**18.7 ± 0.30**	**4.73**
**6**	**34.3 ± 1.13**	**4.46**	**37.6 ± 0.91**	**4.42**
**7**	124.6 ± 1.31	3.90	113.0 ± 0.92	3.95
**8**	81.2 ± 0.63	4.09	**30.6 ± 0.31**	**4.51**
**9**	99.3 ± 0.92	4.00	89.8 ± 0.72	4.05
**10**	>300	-	>300	-
**VII ^[c]^**	>300	-	>300	-
**VIII ^[c]^**	>300	-	>300	-
**IX ^[c]^**	>300	-	>300	-
**X ^[c]^**	>300	-	>300	-
**Doxorubicin**	**0.27 ± 0.08**	**6.57**	**4.07 ± 0.92**	**5.39**

^[a]^ IC_50_ (half-maximal inhibitory concentration) mean ± SD values; ^[b]^ pIC_50_ = −log IC_50_(M); bold: significant cytotoxic effect, IC_50_ < 40 µM; IC_50_ > 300 µM: inactive compounds; ^[c]^: CBHQ compounds (taken from reference [41]).

**Table 2:** Comparison (ΔG*_bin_*, kcal/mol) of synthesized cytotoxic hybrids with kinase proteins overexpressed in cancer and kinase inhibitors approved by the FDA.

Compound	Target Proteins					
	EGFR	MEK1	c-MET	TPK	TRKA	CK4
**4**	−10.7	−9.6	−9.7	−8.6	−9.2	−8.4
**5**	−8.5	−8.5	−9.5	−8.5	−9.2	−8.3
**6**	−11.3	−10.0	−10.3	−10.0	−9.5	−7.9
**7**	−11.4	−10.7	−10.3	−10.4	−10.0	−8.7
**8**	−11.0	−10.3	−9.7	−10.2	−9.3	−8.8
**9**	−11.2	−10.6	−9.9	−10.6	−10.1	−9.0
**P avge.**	**−10.68**	**−9.95**	**−9.90**	**−9.72**	**−9.55**	**−8.52**
**Erlotinib**	−8.6	−8.0	−9.1	−8.5	−8.8	−7.2
**Anlotinib**	−9.2	−9.7	−9.0	−9.0	−10.7	−8.5
**Crizotinib**	−9.7	−9.9	−10.0	−10.2	−10.1	−8.0

Proteins with their respective (PDB) entries. EGFR: epidermal growth factor receptor (5GTY); MEK1: MAPK/ERK kinase (4AN3); c-MET: mesenchymal–epithelial transition factor (3RHK); TPK: tyrosine protein kinase (4EHZ); TRKA: tropomyosin receptor kinase A (6PL2); CK4: cyclin-dependent kinase 4 (1PXL). Bold: Average ΔG*_bin_* value for each protein.

**Table 3:** Predicted binding free-energy values (ΔG*_bin_*, kcal/mol) and binding site contacts of synthesized cytotoxic hybrids with amino acids of EGFR, MEK1, CK4, and TPK.

Compound	ΔG*_bin_*	H Bonds and Hydrophobic Contacts in the Binding Site *
	**EGFR** (mean ΔG*_bin_* = −10.68 kcal/mol)	
**4**	−10.7	Leu718, Gly719, Ser720, Val726 */Ala743 *, Ile744, Lys745 *, Met766, Ala767, Val769, Cys775, Arg776, Leu777, Leu788, Ile789, Met790 */Leu792, Met793, Gly796, Cys797 */Arg841, Leu844, Thr854, **Asp855**, Phe856, Leu858
**5**	−8.5	Leu718, Gly719, Ser720, Gly721, Val726 */Ala743 *, Ile744, Lys745 *, Met766 *, Cys775, Arg776, Leu777 *, Leu788 *, Ile789, Met790 */Gln791, Leu792, Met793, Gly796, Cys797/Asp800, Arg841, Asn842, Leu844, **Thr854**, Asp855, Leu858
**6**	−11.3	Leu718 *, Val726 */Ala743 *, Ile744, Lys745 *, Val 769, Cys775, Met766, Arg776, Leu777, Leu788 *, Ile789, Met790 */Leu792, Met793, Gly796, Cys797 */Asp800, Leu844, Thr854, **Asp855**, Phe856, Leu858
**7**	−11.4	Leu718 *, Val726 */**Ala743** *, Ile744, **Lys745** *, Met766, Val769, Cys775, Arg776, Leu777, **Leu788**, Ile789, Met790 */Leu792, Met793, Gly796, Cys797 *, Asp800, Leu844, Thr854, Asp855, Phe856, Leu858
**8**	−11.0	Leu718, Gly719, Val726 */**Ala743**, Ile744, Lys745 *, Met766, Cys775, Arg776, Leu777, Leu788 *, Ile789, Met790 *,/Leu792, Met793, Gly796, Cys797 */Asp800, Arg841, Leu844, Ile853, Thr854, Asp855, Phe856 *, Leu858
**9**	−11.2	Leu718 *, Gly719, Val726 */Ala743 *, Ile744, Lys745, Met766, Val769, Cys775, Arg776, Leu777, Leu788 *, Ile789, Met790 */Leu792, Met793, Gly796, Cys797 */Asp800, Leu844, Ile853, Thr854, Asp855, Phe856, Leu858
	**MEK1** (mean ΔG*_bin_* = −9.95 kcal/mol)	
**4**	−9.6	Lys97, Ile99, Leu115, Leu118, Cys121/Ile126, Val127, Gly128, Phe129/Ile141, Met143, Arg189 *, Asp190/Leu206, Cys207, Asp208, Phe209, Gly210, **Val211**, Ser212, Leu215 *, Ile216 *, Met219
**5**	−8.5	Asn78, Lys97, Ile99, Leu115, Leu118 */Ile141, Met143 *, His188, Arg189, Asp190/Cys207, Asp208, Phe209 *, Gly210, Val211, Ser212, Leu215, Ile216, Met219 */Ala220, Gly225, Thr226, Met230, Arg234
**6**	−10.0	Lys97, Leu115, Leu118 */Ile126, Val127, Gly128, Phe129/Ile141, Met143 *, Arg189, Asp190/Leu206, Cys207 *, Asp208 *, Phe209 *, Gly210, Val211, Ser212, Leu215 *, Ile216, Met219/Ala220, Gly225, Thr226, Met230, Arg234
**7**	−10.7	Lys97, Leu115, Leu118 */Ile126, Val127, Gly128, Phe129/Ile141, Met143 *, Glu144, Arg189, Asp190/Leu206, Cys207 *, Asp208, Phe209, Gly210, Val211, **Ser212**, Leu215 *, Ile216, Met219/Ala220, Thr226, Met230, Arg234
**8**	−10.3	**Lys97**, Ile99, Leu115, Leu118 */Val127, Gly128, Phe129/Ile141 *, Met143 *, Arg189, Asp190/Cys207, Asp208 *, Phe209 *, Gly210, Val211, Leu215, Ile216, Met219 */Ala220, Met230, Arg234, Tyr240
**9**	−10.6	**Lys97**, Ile99, Leu115, Leu118 */Ile126, Val127, Gly128, Phe129/Ile141 *, Met143 *, Arg189, Asp190/Leu206, Cys207 *, Asp208 *, Phe209 *, Gly210, Val211, Ser212, Leu215 *, Ile216, Met219 */Ala220, Met230, Arg234, Tyr240
**c-MET** (mean ΔG*_bin_* = −9.82 kcal/mol)		
**4**	−9.7	Ile1084, Gly1085, His1088, Phe1089 *, Val1092/Ala1108, Val1109, Lys1110, Val1155, Leu1157, Gly1163, Asp1164, **Asn1167/**Arg1208, Met1211, Phe1223, Ala1226, Arg1227 *, Met1229/Tyr1230, Asp1231, Tyr1234, Tyr1235
**5**	−9.5	Ile1084, Gly1085, Gly1087, His1088, Phe1089, Val1092/Ala1108, Leu1140, Leu1157, Gly1163, Asp1164 *, **Asn1167**/Arg1208, Met1211, Phe1223, **Arg1227** *, Met1229/Tyr1230, Asp1231, Tyr1234, Tyr1235
**6**	−10.3	Ile1084, Gly1085, Phe1089 *, Val1092/Ala1108, Lys1110, Val1155, Leu1157, His1162, Gly1163, Asp1164, Asn1167/Phe1168, Asn1171, His1174, Arg1208, Met1211, Phe1223, Ala1226, Arg1227
**7**	−10.3	Gly1085, Phe1089 *, Val1092, Ala1108, Lys1110, Leu1112, Val1155, Leu1157, His1162, Gly1163, Asp1164, Asn1167, Phe1168, Asn1171, His1174, Arg1208, Met1211, Phe1223, Ala1226, Arg1227
**8**	−9.7	Ile1084, Phe1089 *, Val1092/Ala1108, Leu1140, Leu1157, Pro1158, Tyr1159, Met1160, Gly1163, Asp1164, **Arg1166**, Asn1167 */Asn1208 *, Met1211, Phe1223, **Arg1227/Tyr1234**
**9**	−9.9	Gly1087, His1088, Phe1089, Val1092 */Ala1108 *, Lys1110 *, Leu1112, Val1155, Leu1157 *, Asp1164 *, Arg1166/Asp1167, Arg1208, Met1211, Phe1223 *, Ala1226, **Arg1227**, Met1229/Tyr1230, Asp1231, Tyr1234, Tyr1235

Bolded names correspond to amino acids involved in H bonds with the corresponding synthesized cytotoxic hybrids. Partial interacting peptide sequences are separated and differentiated by colors to facilitate comparisons of similar interactions. Residues with * correspond to amino acids that interact with the ligand through any type of Pi interaction.

**Table 4:** Binding site contacts of compound **7**, ligands, and drugs into EGFR, MEK1, and c-MET.

Compound	ΔG*_bin_* (kcal/mol)	H Bonds and Hydrophobic Contacts in the Binding Site
		EGFR
**7**	−11.4	Leu718, Val726, **Ala743**, Ile744, **Lys745**, Met766, Val769, Cys775, Arg776, Leu777, **Leu788**, Ile789, Met790, Leu792, Met793, Gly796, Cys797, Asp800, Leu844, Thr854, Asp855, Phe856, Leu858
**Ligand 1 ^[a]^**	−10.7	Leu718, Gly719, **Ser720**, Val726, Ala743, Ile744, Lys745, Met766, Cys775, Arg776, Leu777, Leu788, Ile789, Met790, Gln791, Leu792, Met793, Gly796, Cys797, Asp800, Arg841, Leu844, Thr854, Asp855, Phe856, Leu858
**Erlotinib ^[b]^**	−8.6	Leu718, Gly721, Val726, Ala743, Lys745, Met766, Cys775, Arg776, Leu777, Leu788, Met790, Met793, Gly796, **Arg841**, Arg842, Leu844, Thr854, Asp855, Phe856, Leu858
		**MEK1**
**7**	−10.7	Lys97, Leu115, Leu118, Ile126, Val127, Gly128, Phe129, Ile141, Met143, Glu144, Arg189, Asp190, Leu206, Cys207, Asp208, Phe209, Gly210, Val211, **Ser212**, Leu215, Ile216, Met219, Ala220, Thr226, Met230, Arg234
**Ligand 2 ^[a]^**	−11.4	Asn78, Gly79, **Lys97**, Ile99, Leu115, Leu118, Val127, Phe129, Ile141, Met143, Asp190, Cys207, Asp208, Phe209, Gly210, Val211, **Ser212**, Leu215, Ile216, Met219
**Anlotinib ^[b]^**	−9.7	Lys97, Leu115, Leu118, Val127, Gly128, Phe129, Ile141, Met143, Arg189, Asp190, Cys207, Asp208, Phe209, Gly210, Val211, Ser212, Leu215, Ile216, Met219, Gly225
		**c-MET**
**7**	−10.3	Gly1085, Phe1089, Val1092, Ala1108, Lys1110, Leu1112, Val1155, Leu1157, His1162, Gly1163, Asp1164, Asn1167, Phe1168, Asn1171, His1174, Arg1208, Met1211, Phe1223, Ala1226, Arg1227
**Ligand 3 ^[a]^**	−14.6	Ile1084, Gly1085, Phe1089, Val1092, Ala1108, Lys1110, Leu1140, Leu1157, **Met1158**, Tyr1159, **Met1160**, Gly1163, Met1211, Phe1223, Ala1226, Arg1227
**Crizotinib ^[b]^**	−10.0	Ile1084, Gly1085, Phe1089, Val1092, Ala1108, Lys1110, Arg1227, Leu1157, Asp1164, Arg1166, Asn1167, Arg1208, Met1211, Phe1223, Ala1226, Tyr1234

^[a]^ Ligands 1, 2, and 3 correspond to 1-[(3R)-3-[4-azanyl-3-[3-chloranyl-4-[(6-methylpyridin-2-yl)methoxy]phenyl]pyrazolo [3,4-d]pyrimidin-1-yl]piperidin-1-yl]prop-2-en-1-one, N-[(2S)-2,3-bis(oxidanyl)propoxy]-3,4-bis(fluoranyl)-2-[(2-fluoranyl-4-iodanyl-phenyl)amino]benzamide, and 2-methyl-1-(piperidin-4-yl)-1,6-dihydroimidazo [4,5-d]pyrrolo [2,3-b]pyridine, respectively. Three-dimensional structures of ligands 1, 2, and 3 were extracted from the Protein Data Bank using PDB IDs 5GTY, 4AN3, and 3RHK, respectively. ^[b]^ Drug compounds that act as inhibitors of biological targets. Words colored in blue, green, and red correspond to amino acids shared by **7** and ligands; **7** and drugs; and **7**, ligands, and drugs, respectively. Bolded names correspond to amino acids involved in H bonds and 7–enzyme interactions.

**Table 5:** Comparison of predicted toxicity risks ^a^ and drug-likeness scores ^a^ for hybrids **4**–**9**.

Compound	M	T	I	R	Drug Likeness
**4**	h	n	n	n	−1.58
**5**	h	n	h	n	−1.49
**6**	n	n	n	n	−0.47
**7**	n	n	n	n	**0.53**
**8**	n	n	n	n	**0.83**
**9**	n	n	n	n	−0.72

^a^ Predicted by DataWarrior algorithm; M: mutagenic; T: tumorigenic; I: irritant; R: reproductive effect. Levels—n: none; h: high.

## Data Availability

Data is contained within the article and Supplementary Materials.

## Supplementary Materials

The following supporting information can be downloaded at: https://www.mdpi.com/article/10.3390/ijms25137281/s1.

## References

[1] Rebucci M., Michiels C. (2013). Molecular Aspects of Cancer Cell Resistance to Chemotherapy. Biochem. Pharmacol..

[2] Patel T.K., Adhikari N., Amin S.A., Biswas S., Jha T., Ghosh B. (2021). Small Molecule Drug Conjugates (SMDCs): An Emerging Strategy for Anticancer Drug Design and Discovery. New J. Chem..

[3] Bertanha C., Januário A., Alvarenga T., Pimenta L., Silva M., Cunha W., Pauletti P. (2014). Quinone and Hydroquinone Metabolites from the Ascidians of the Genus Aplidium. Mar. Drugs.

[4] Giner R.M., Ríos J.L., Máñez S. (2022). Antioxidant Activity of Natural Hydroquinones. Antioxidants.

[5] Lane A.L., Mular L., Drenkard E.J., Shearer T.L., Engel S., Fredericq S., Fairchild C.R., Prudhomme J., Le Roch K., Hay M.E. (2010). Ecological Leads for Natural Product Discovery: Novel Sesquiterpene Hydroquinones from the Red Macroalga *Peyssonnelia* sp.. Tetrahedron.

[6] Scozzafava A., Kalın P., Supuran C.T., Gülçin İ., Alwasel S.H. (2015). The Impact of Hydroquinone on Acetylcholine Esterase and Certain Human Carbonic Anhydrase Isoenzymes (HCA I, II, IX, and XII). J. Enzym. Inhib. Med. Chem..

[7] Tasseau O., Mosset P., Barillé-Nion S., Gautier F., Juin P., Levoin N., Amireddy N., Kalivendi S., Grée R. (2018). Synthesis and Cytotoxicity Studies of Newly Designed Benzyl-Hydroquinone Derivatives. Med. Chem. Res..

[8] Sladic D., Gasic M. (2006). Reactivity and Biological Activity of the Marine Sesquiterpene Hydroquinone Avarol and Related Compounds from Sponges of the Order Dictyoceratida. Molecules.

[9] Peduto A., Scuotto M., Krauth V., Roviezzo F., Rossi A., Temml V., Esposito V., Stuppner H., Schuster D., D’Agostino B. (2017). Optimization of Benzoquinone and Hydroquinone Derivatives as Potent Inhibitors of Human 5-Lipoxygenase. Eur. J. Med. Chem..

[10] Chang H.-S., Lin Y.-J., Lee S.-J., Yang C.-W., Lin W.-Y., Tsai I.-L., Chen I.-S. (2009). Cytotoxic Alkyl Benzoquinones and Alkyl Phenols from Ardisia Virens. Phytochemistry.

[11] Byeon S., Yi Y.-S., Lee J., Yang W., Kim J., Kim J., Hong S., Kim J.-H., Cho J. (2018). Hydroquinone Exhibits In Vitro and In Vivo Anti-Cancer Activity in Cancer Cells and Mice. Int. J. Mol. Sci..

[12] Sunassee S.N., Davies-Coleman M.T. (2012). Cytotoxic and Antioxidant Marine Prenylated Quinones and Hydroquinones. Nat. Prod. Rep..

[13] Müller W.E., Sladić D., Zahn R.K., Bässler K.H., Dogović N., Gerner H., Gasić M.J., Schröder H.C. (1987). Avarol-Induced DNA Strand Breakage in Vitro and in Friend Erythroleukemia Cells. Cancer Res..

[14] Matulja D., Vranješević F., Kolympadi Markovic M., Pavelić S.K., Marković D. (2022). Anticancer Activities of Marine-Derived Phenolic Compounds and Their Derivatives. Molecules.

[15] Yoo H.-D., Leung D., Sanghara J., Daley D., Soest R., Andersen R.J. (2003). Isoarenarol, A New Protein Kinase Inhibitor from the Marine Sponge Dysidea Arenaria. Pharm. Biol..

[16] Nursamsiar, Asnawi A., Kartasasmita R.E., Ibrahim S., Tjahjono D.H. (2018). Synthesis, Biological Evaluation, and Docking Analysis of Methyl Hydroquinone and Bromo Methyl Hydroquinone as Potent Cyclooxygenase (COX-1 and COX-2) Inhibitors. J. Appl. Pharm. Sci..

[17] Rammohan A., Reddy J.S., Sravya G., Rao C.N., Zyryanov G.V. (2020). Chalcone Synthesis, Properties and Medicinal Applications: A Review. Environ. Chem. Lett..

[18] Al Zahrani N.A., El-Shishtawy R.M., Elaasser M.M., Asiri A.M. (2020). Synthesis of Novel Chalcone-Based Phenothiazine Derivatives as Antioxidant and Anticancer Agents. Molecules.

[19] Pereira R., Silva A.M.S., Ribeiro D., Silva V.L.M., Fernandes E. (2023). Bis-Chalcones: A Review of Synthetic Methodologies and Anti-Inflammatory Effects. Eur. J. Med. Chem..

[20] Rajendran G., Bhanu D., Aruchamy B., Ramani P., Pandurangan N., Bobba K.N., Oh E.J., Chung H.Y., Gangadaran P., Ahn B.-C. (2022). Chalcone: A Promising Bioactive Scaffold in Medicinal Chemistry. Pharmaceuticals.

[21] Liu W., He M., Li Y., Peng Z., Wang G. (2022). A Review on Synthetic Chalcone Derivatives as Tubulin Polymerisation Inhibitors. J. Enzym. Inhib. Med. Chem..

[22] Ouyang Y., Li J., Chen X., Fu X., Sun S., Wu Q. (2021). Chalcone Derivatives: Role in Anticancer Therapy. Biomolecules.

[23] WalyEldeen A.A., Sabet S., El-Shorbagy H.M., Abdelhamid I.A., Ibrahim S.A. (2023). Chalcones: Promising Therapeutic Agents Targeting Key Players and Signaling Pathways Regulating the Hallmarks of Cancer. Chem. Biol. Interact..

[24] Constantinescu T., Lungu C.N. (2021). Anticancer Activity of Natural and Synthetic Chalcones. Int. J. Mol. Sci..

[25] Mezgebe K., Melaku Y., Mulugeta E. (2023). Synthesis and Pharmacological Activities of Chalcone and Its Derivatives Bearing N -Heterocyclic Scaffolds: A Review. ACS Omega.

[26] Mathew B., Suresh J., Anbazghagan S., Paulraj J., Krishnan G.K. (2014). Heteroaryl Chalcones: Mini Review about Their Therapeutic Voyage. Biomed. Prev. Nutr..

[27] Farhat J., Alzyoud L., Alwahsh M., Al-Omari B. (2022). Structure–Activity Relationship of Benzofuran Derivatives with Potential Anticancer Activity. Cancers.

[28] Gao F., Huang G., Xiao J. (2020). Chalcone Hybrids as Potential Anticancer Agents: Current Development, Mechanism of Action, and Structure-activity Relationship. Med. Res. Rev..

[29] Ardiansah B. (2019). Chalcones Bearing N, O, and S-Heterocycles: Recent Notes on Their Biological Significances. J. Appl. Pharm. Sci..

[30] Haider K., Shafeeque M., Yahya S., Yar M.S. (2022). A Comprehensive Review on Pyrazoline Based Heterocyclic Hybrids as Potent Anticancer Agents. Eur. J. Med. Chem. Rep..

[31] Alkamaly O.M., Altwaijry N., Sabour R., Harras M.F. (2021). Dual EGFR/VEGFR2 Inhibitors and Apoptosis Inducers: Synthesis and Antitumor Activity of Novel Pyrazoline Derivatives. Arch. Pharm..

[32] Ahmed M.F., Santali E.Y., El-Haggar R. (2021). Novel Piperazine–Chalcone Hybrids and Related Pyrazoline Analogues Targeting VEGFR-2 Kinase; Design, Synthesis, Molecular Docking Studies, and Anticancer Evaluation. J. Enzym. Inhib. Med. Chem..

[33] Dofe V.S., Sarkate A.P., Tiwari S.V., Lokwani D.K., Karnik K.S., Kale I.A., Dodamani S., Jalalpure S.S., Burra P.V.L.S. (2020). Ultrasound Assisted Synthesis of Tetrazole Based Pyrazolines and Isoxazolines as Potent Anticancer Agents via Inhibition of Tubulin Polymerization. Bioorg. Med. Chem. Lett..

[34] Song Y., Feng S., Feng J., Dong J., Yang K., Liu Z., Qiao X. (2020). Synthesis and Biological Evaluation of Novel Pyrazoline Derivatives Containing Indole Skeleton as Anti-Cancer Agents Targeting Topoisomerase II. Eur. J. Med. Chem..

[35] Singh A.K., Kumar A., Singh H., Sonawane P., Paliwal H., Thareja S., Pathak P., Grishina M., Jaremko M., Emwas A.-H. (2022). Concept of Hybrid Drugs and Recent Advancements in Anticancer Hybrids. Pharmaceuticals.

[36] Mancini I., Vigna J., Sighel D., Defant A. (2022). Hybrid Molecules Containing Naphthoquinone and Quinolinedione Scaffolds as Antineoplastic Agents. Molecules.

[37] Molinari A., Oliva A., Arismendi M., Imbarack E., Gálvez C., Maldonado J., Feliciano A.S. (2015). The Synthesis of Some Fused Pyrazolo-1,4-Naphthoquinones. J. Heterocycl. Chem..

[38] Maldonado J., Acevedo W., Molinari A., Oliva A., Knox M., San Feliciano A. (2023). Synthesis, in Vitro Evaluation and Molecular Docking Studies of Novel Naphthoisoxazolequinone Carboxamide Hybrids as Potential Antitumor Agents. Polycycl. Aromat. Compd..

[39] Molinari A., Oliva A., Arismendi-Macuer M., Guzmán L., Fuentealba M., Knox M., Vinet R., San Feliciano A. (2015). New 1H-Benzo[f]Indazole-4,9-Diones Conjugated with C-Protected Amino Acids and Other Derivatives: Synthesis and in Vitro Antiproliferative Evaluation. Molecules.

[40] Molinari A., Oliva A., Arismendi-Macuer M., Guzmán L., Acevedo W., Aguayo D., Vinet R., San Feliciano A. (2019). Antiproliferative Benzoindazolequinones as Potential Cyclooxygenase-2 Inhibitors. Molecules.

[41] Maldonado J., Oliva A., Molinari A., Acevedo W. (2023). 2-Acetyl-5,8-Dihydro-6-(4-Methyl-3-Pentenyl)-1,4-Naphthohydroquinone-Derived Chalcones as Potential Anticancer Agents. Molecules.

[42] Guo S., Colbert L.S., Fuller M., Zhang Y., Gonzalez-Perez R.R. (2010). Vascular Endothelial Growth Factor Receptor-2 in Breast Cancer. Biochim. Biophys. Acta-Rev. Cancer.

[43] Ali S., Coombes R.C. (2000). Estrogen Receptor Alpha in Human Breast Cancer: Occurrence and Significance. J. Mammary Gland Biol. Neoplasia.

[44] Kim J., Kim S., Ko S., In Y., Moon H., Ahn S.K., Kim M.K., Lee M., Hwang J., Ju Y.S. (2015). Recurrent Fusion Transcripts Detected by Whole-transcriptome Sequencing of 120 Primary Breast Cancer Samples. Genes Chromosom. Cancer.

[45] Ahmad D.A.J., Negm O.H., Alabdullah M.L., Mirza S., Hamed M.R., Band V., Green A.R., Ellis I.O., Rakha E.A. (2016). Clinicopathological and Prognostic Significance of Mitogen-Activated Protein Kinases (MAPK) in Breast Cancers. Breast Cancer Res. Treat..

[46] Pashirzad M., Khorasanian R., Fard M.M., Arjmand M.-H., Langari H., Khazaei M., Soleimanpour S., Rezayi M., Ferns G.A., Hassanian S.M. (2021). The Therapeutic Potential of MAPK/ERK Inhibitors in the Treatment of Colorectal Cancer. Curr. Cancer Drug Targets.

[47] Sharma B., Singh V.J., Chawla P.A. (2021). Epidermal Growth Factor Receptor Inhibitors as Potential Anticancer Agents: An Update of Recent Progress. Bioorg. Chem..

[48] Nawaz F., Alam O., Perwez A., Rizvi M.A., Naim M.J., Siddiqui N., Ul Firdaus J., Rahman S., Jha M., Sheikh A.A. (2020). Design, Synthesis, Molecular Docking, and Anticancer Evaluation of Pyrazole Linked Pyrazoline Derivatives with Carbothioamide Tail as EGFR Kinase Inhibitors. Anticancer Agents Med. Chem..

[49] Raghu M.S., Pradeep Kumar C.B., Prashanth M.K., Yogesh Kumar K., Prathibha B.S., Kanthimathi G., Alissa S.A., Alghulikah H.A., Osman S.M. (2021). Novel 1,3,5-Triazine-Based Pyrazole Derivatives as Potential Antitumor Agents and EFGR Kinase Inhibitors: Synthesis, Cytotoxicity, DNA Binding, Molecular Docking and DFT Studies. New J. Chem..

[50] Al-Anazi M., Khairuddean M., Al-Najjar B.O., Murwih Alidmat M., Nur Syazni Nik Mohamed Kamal N., Muhamad M. (2022). Synthesis, Anticancer Activity and Docking Studies of Pyrazoline and Pyrimidine Derivatives as Potential Epidermal Growth Factor Receptor (EGFR) Inhibitors. Arab. J. Chem..

[51] Abourehab M.A.S., Alqahtani A.M., Youssif B.G.M., Gouda A.M. (2021). Globally Approved EGFR Inhibitors: Insights into Their Syntheses, Target Kinases, Biological Activities, Receptor Interactions, and Metabolism. Molecules.

[52] Ge X., Zhang Y., Huang F., Wu Y., Pang J., Li X., Fan F., Liu H., Li S. (2021). EGFR Tyrosine Kinase Inhibitor Almonertinib Induces Apoptosis and Autophagy Mediated by Reactive Oxygen Species in Non-Small Cell Lung Cancer Cells. Hum. Exp. Toxicol..

[53] Shen G., Zheng F., Ren D., Du F., Dong Q., Wang Z., Zhao F., Ahmad R., Zhao J. (2018). Anlotinib: A Novel Multi-Targeting Tyrosine Kinase Inhibitor in Clinical Development. J. Hematol. Oncol..

[54] Hu H., Liu Y., Tan S., Xie X.X., He J., Luo F., Wang L. (2020). Anlotinib Exerts Anti-Cancer Effects on KRAS-Mutated Lung Cancer Cell Through Suppressing the MEK/ERK Pathway. Cancer Manag. Res..

[55] Rodig S.J., Shapiro G.I. (2010). Crizotinib, a Small-Molecule Dual Inhibitor of the c-Met and ALK Receptor Tyrosine Kinases. Curr. Opin. Investig. Drugs.

[56] Tang P.A., Tsao M.-S., Moore M.J. (2006). A Review of Erlotinib and Its Clinical Use. Expert Opin. Pharmacother..

[57] Neal J.W., Dahlberg S.E., Wakelee H.A., Aisner S.C., Bowden M., Huang Y., Carbone D.P., Gerstner G.J., Lerner R.E., Rubin J.L. (2016). Erlotinib, Cabozantinib, or Erlotinib plus Cabozantinib as Second-Line or Third-Line Treatment of Patients with EGFR Wild-Type Advanced Non-Small-Cell Lung Cancer (ECOG-ACRIN 1512): A Randomised, Controlled, Open-Label, Multicentre, Phase 2 Trial. Lancet Oncol..

[58] Tsao M.-S., Sakurada A., Cutz J.-C., Zhu C.-Q., Kamel-Reid S., Squire J., Lorimer I., Zhang T., Liu N., Daneshmand M. (2005). Erlotinib in Lung Cancer—Molecular and Clinical Predictors of Outcome. N. Engl. J. Med..

[59] Bhangoo M.S., Sigal D. (2019). TRK Inhibitors: Clinical Development of Larotrectinib. Curr. Oncol. Rep..

[60] Federman N., McDermott R. (2019). Larotrectinib, a Highly Selective Tropomyosin Receptor Kinase (TRK) Inhibitor for the Treatment of TRK Fusion Cancer. Expert Rev. Clin. Pharmacol..

[61] Zhang X., Li B., Song M., Song J. (2004). Expression and Significance of ERK Protein in Human Breast Carcinoma. Chin. J. Cancer Res..

[62] Ho-Yen C.M., Jones J.L., Kermorgant S. (2015). The Clinical and Functional Significance of C-Met in Breast Cancer: A Review. Breast Cancer Res..

[63] Griffin N., Marsland M., Roselli S., Oldmeadow C., Attia J., Walker M.M., Hondermarck H., Faulkner S. (2020). The Receptor Tyrosine Kinase TrkA Is Increased and Targetable in HER2-Positive Breast Cancer. Biomolecules.

[64] Brylinski M. (2018). Aromatic Interactions at the Ligand–Protein Interface: Implications for the Development of Docking Scoring Functions. Chem. Biol. Drug Des..

[65] Molinari A., Oliva A., del Corral J.M.M., Castro M.A., Araya C., García-Grávalos M.D., San Feliciano A. (2004). Cytotoxic–Antineoplastic Activity of Acetyl Derivatives of Prenylnaphthohydroquinone. Il Farm..

[66] Sander T., Freyss J., von Korff M., Rufener C. (2015). DataWarrior: An Open-Source Program For Chemistry Aware Data Visualization And Analysis. J. Chem. Inf. Model..

[67] Jorgensen W.L., Duffy E.M. (2002). Prediction of Drug Solubility from Structure. Adv. Drug Deliv. Rev..

[68] Berman H.M., Battistuz T., Bhat T.N., Bluhm W.F., Bourne P.E., Burkhardt K., Feng Z., Gilliland G.L., Iype L., Jain S. (2002). The Protein Data Bank. Acta Crystallogr. Sect. D Biol. Crystallogr..

[69] Negi R.R., Rana S.V., Gupta V., Gupta R., Chadha V.D., Prasad K.K., Dhawan D.K. (2019). Over-Expression of Cyclooxygenase-2 in Colorectal Cancer Patients. Asian Pac. J. Cancer Prev..

[70] Sylvester P.W. (2014). Targeting Met Mediated Epithelial-mesenchymal Transition in the Treatment of Breast Cancer. Clin. Transl. Med..

[71] Wang Y., Zhou B.P. (2011). Epithelial-Mesenchymal Transition in Breast Cancer Progression and Metastasis. Chin. J. Cancer.

[72] Lazaro G., Smith C., Goddard L., Jordan N., McClelland R., Barrett-Lee P., Nicholson R.I., Hiscox S. (2013). Targeting Focal Adhesion Kinase in ER+/HER2+ Breast Cancer Improves Trastuzumab Response. Endocr. Relat. Cancer.

[73] Nakano M., Fukami T., Gotoh S., Nakajima M. (2017). A-to-I RNA Editing Up-Regulates Human Dihydrofolate Reductase in Breast Cancer. J. Biol. Chem..

[74] Lei H., Deng C.-X. (2017). Fibroblast Growth Factor Receptor 2 Signaling in Breast Cancer. Int. J. Biol. Sci..

[75] Haldosén L.-A., Zhao C., Dahlman-Wright K. (2014). Estrogen Receptor Beta in Breast Cancer. Mol. Cell. Endocrinol..

[76] Chen H., Zhu G., Li Y., Padia R.N., Dong Z., Pan Z.K., Liu K., Huang S. (2009). Extracellular Signal–Regulated Kinase Signaling Pathway Regulates Breast Cancer Cell Migration by Maintaining Slug Expression. Cancer Res..

[77] Sultan A.S., Brim H., Sherif Z.A. (2008). Co-overexpression of Janus Kinase 2 and Signal Transducer and Activator of Transcription 5a Promotes Differentiation of Mammary Cancer Cells through Reversal of Epithelial–Mesenchymal Transition. Cancer Sci..

[78] Trott O., Olson A.J. (2010). AutoDock Vina: Improving the Speed and Accuracy of Docking with a New Scoring Function, Efficient Optimization, and Multithreading. J. Comput. Chem..

[79] Acevedo W., González-Nilo F., Agosin E. (2016). Docking and Molecular Dynamics of Steviol Glycoside–Human Bitter Receptor Interactions. J. Agric. Food Chem..

[80] Humphrey W., Dalke A., Schulten K. (1996). VMD: Visual Molecular Dynamics. J. Mol. Graph..

[81] Dassault Syst èmes BIOVIA (2019). Discovery Studio Visualizer.

