# Supplementary material for: Synthesis, Anticancer Activity, and Docking Studies of Novel Hydroquinone-Chalcone-Pyrazoline Hybrid Derivatives
Source: Int J Mol Sci. 2024 Jul 2;25(13):7281. doi: 10.3390/ijms25137281 (PMC11242894; doi:10.3390/ijms25137281)
Supplement: Supplementary file 1 [file ijms-25-07281-s001.zip › ijms-3073398-supplementary.pdf]

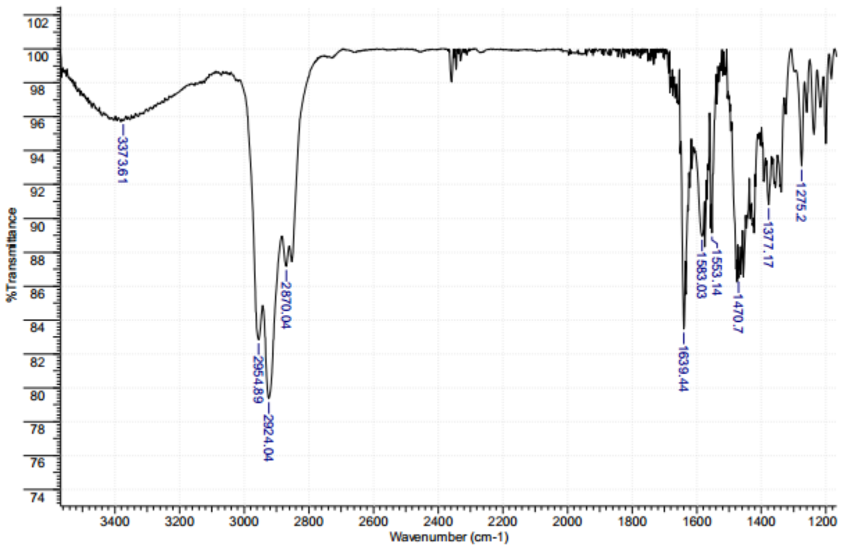

Figure S1. IR spectrum of compound **3**.

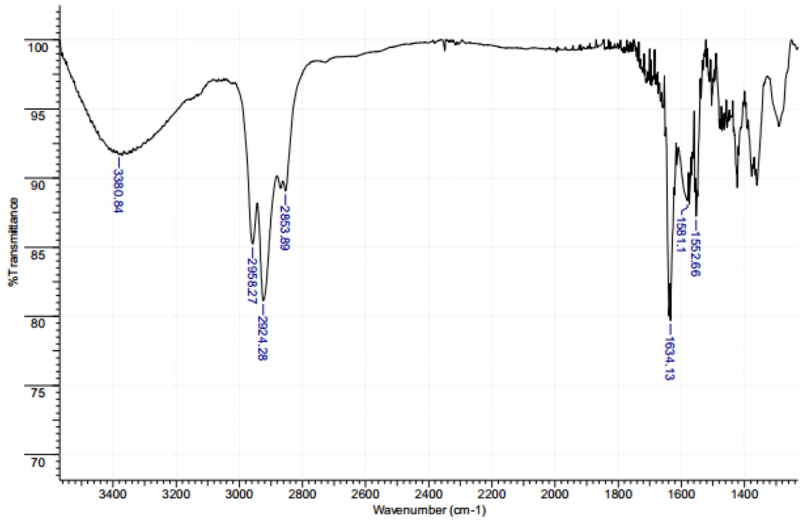

Figure S2. IR spectrum of compound **4**.

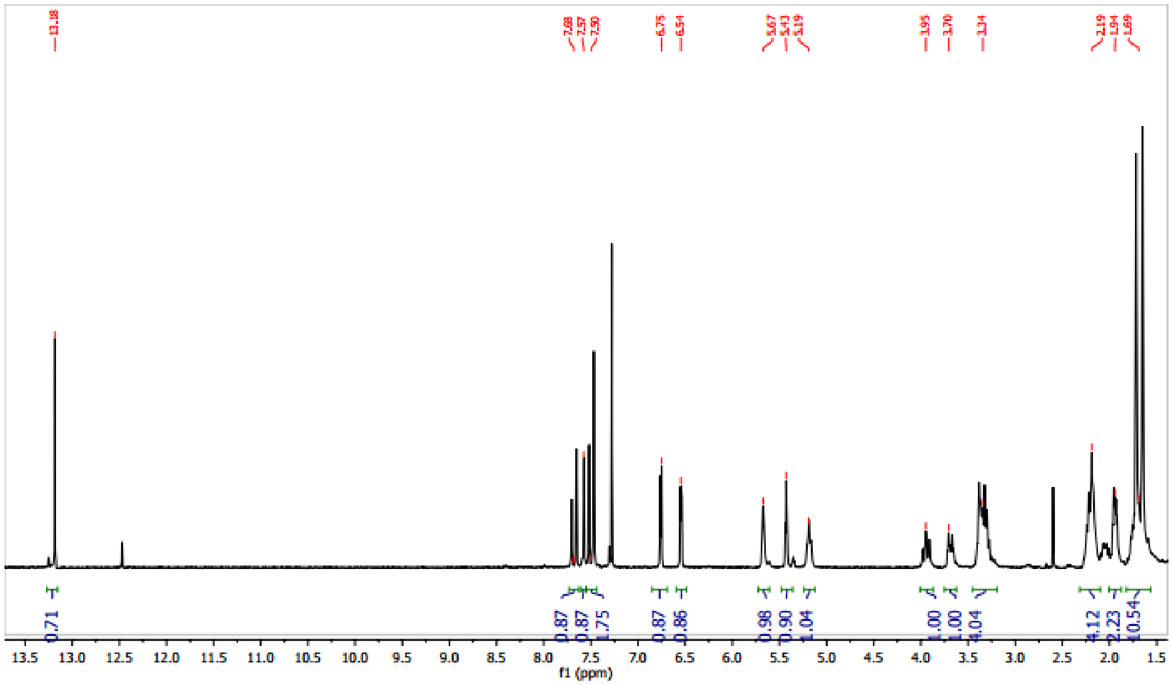

Figure S3. ^{1}H NMR spectrum of compound **3**.

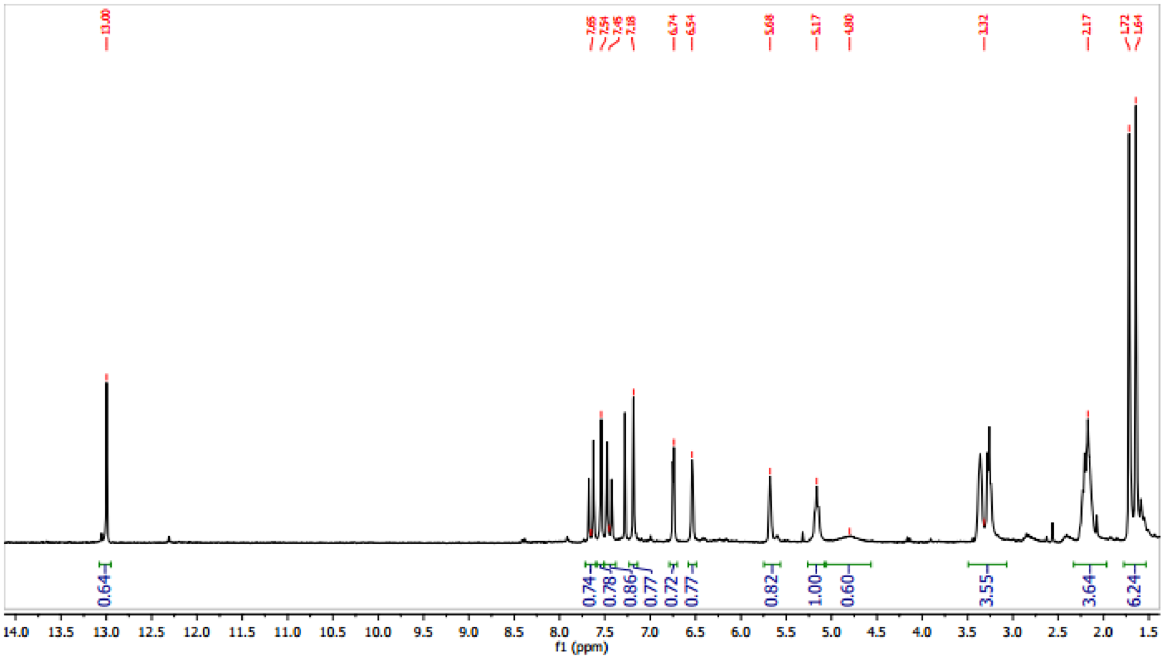

Figure S4. ^{1}H NMR spectrum of compound **4**.

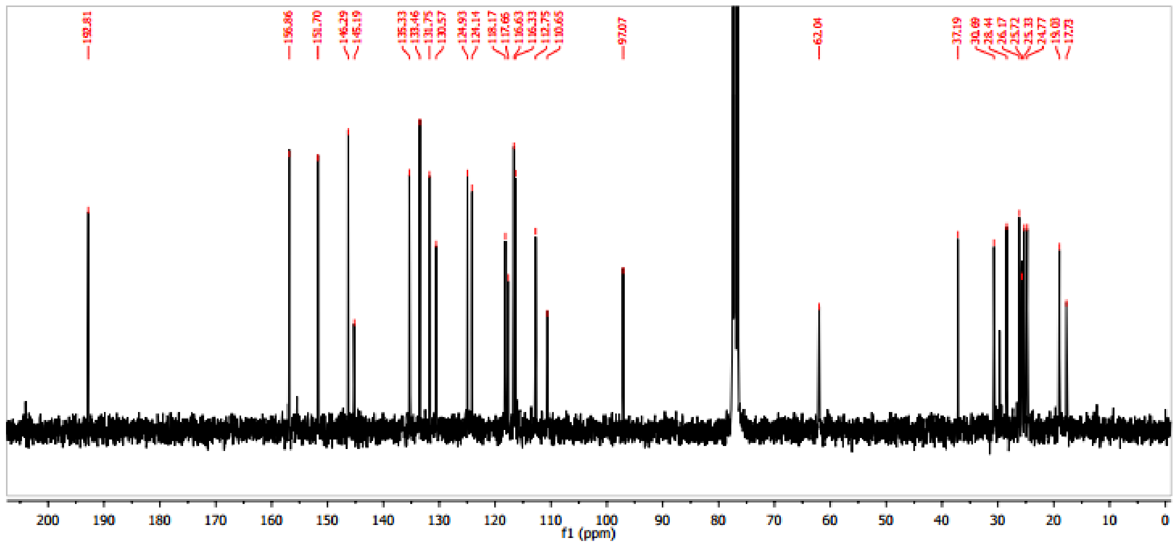

Figure S5. ^{13}C NMR spectrum of compound **3**.

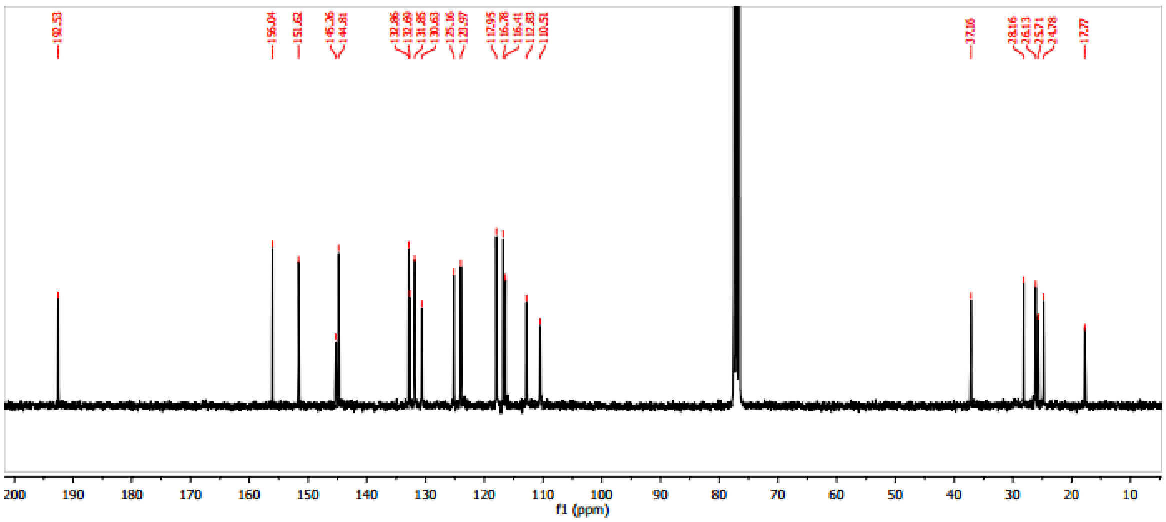

Figure S6. ^{13}C NMR spectrum of compound **4**.

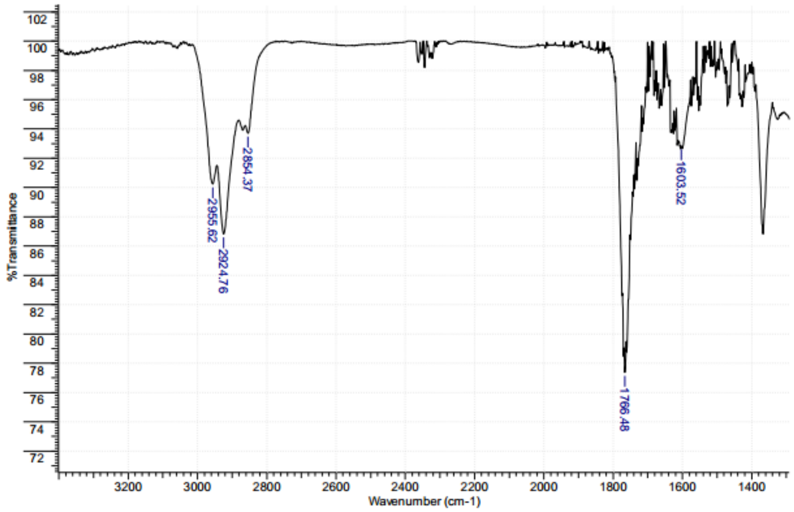

Figure S7. IR spectrum of compound **5**.

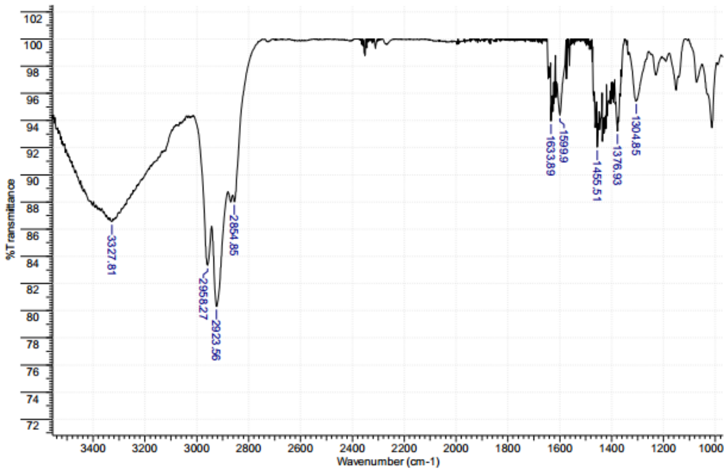

Figure S8. IR spectrum of compound **6**.

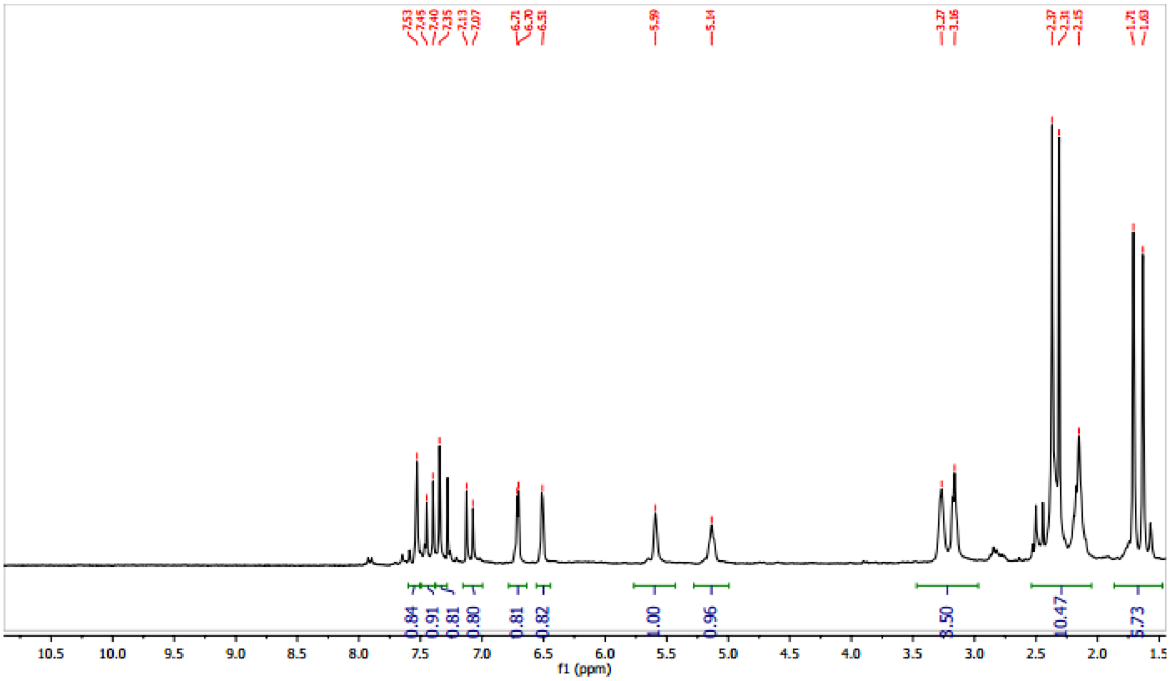

Figure S9. ^{1}H NMR spectrum of compound **5**.

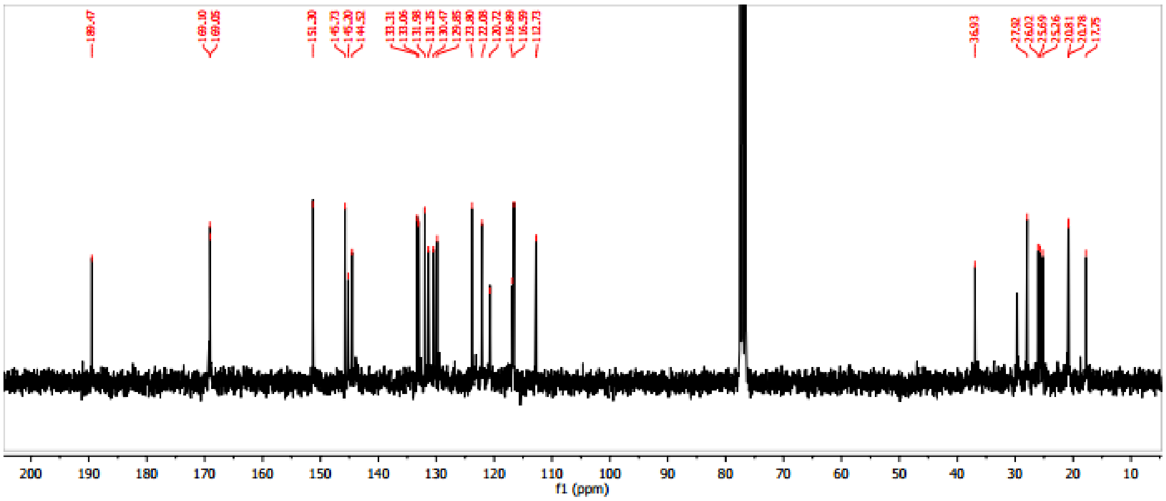

Figure S10. ^{13}C NMR spectrum of compound **5**.

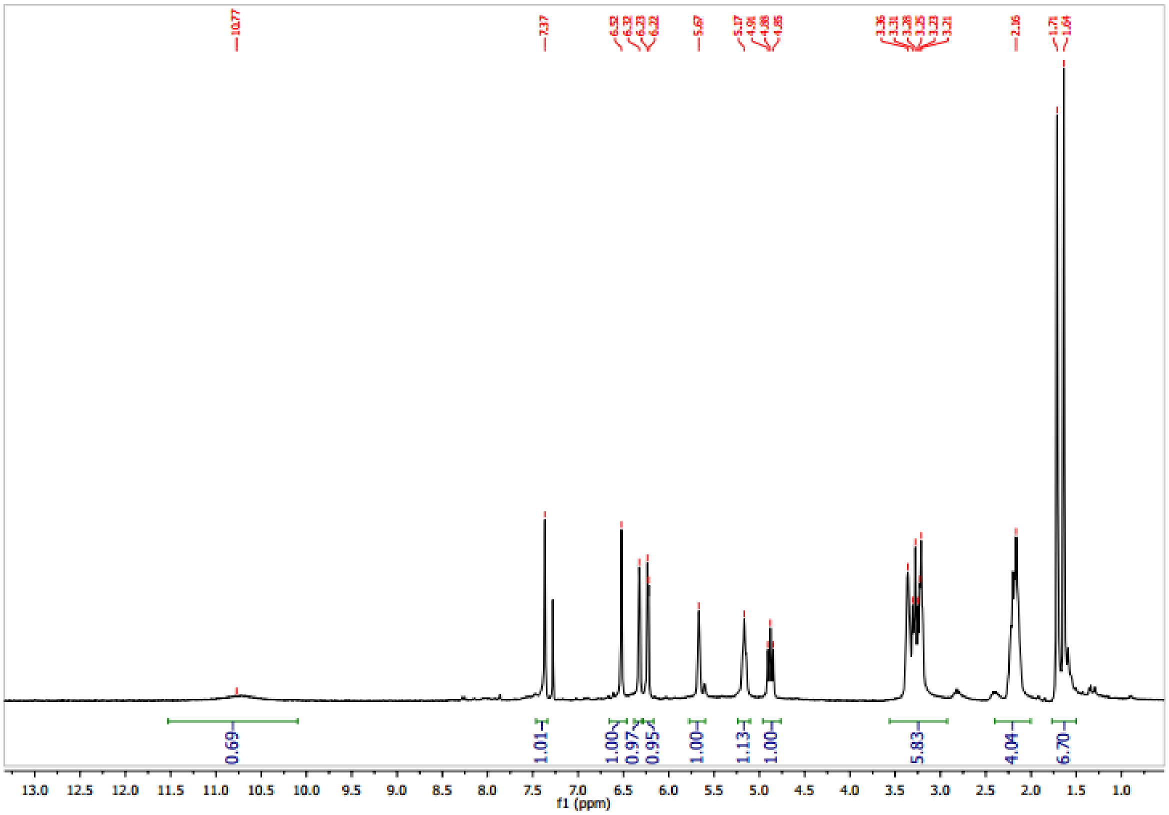

Figure S11. ^{1}H NMR spectrum of compound **6**.

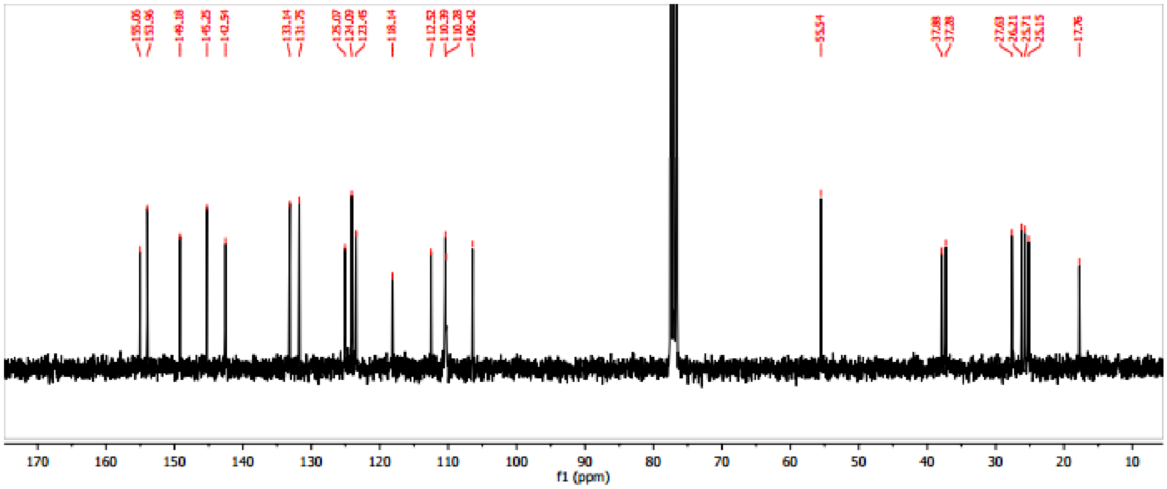

Figure S12. ^{13}C NMR spectrum of compound **6**.

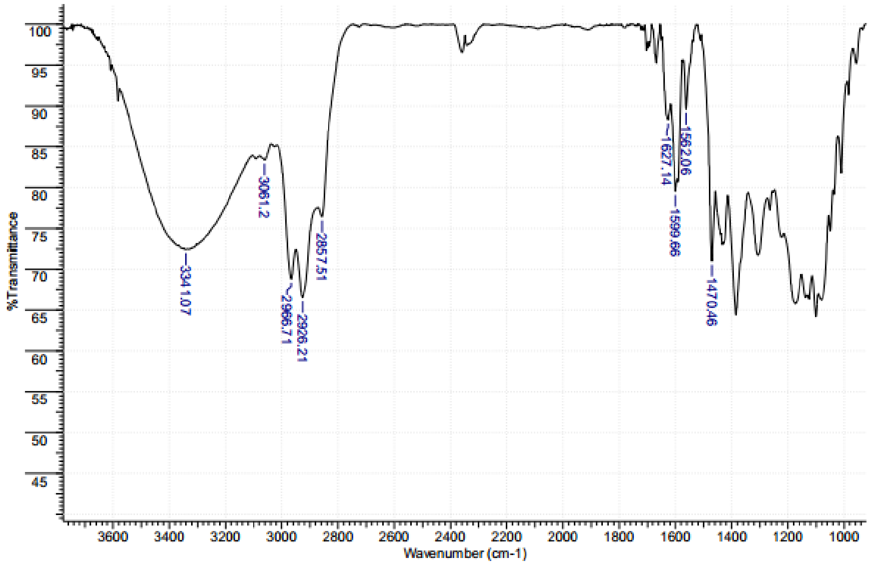

Figure S13. IR spectrum of compound **7**.

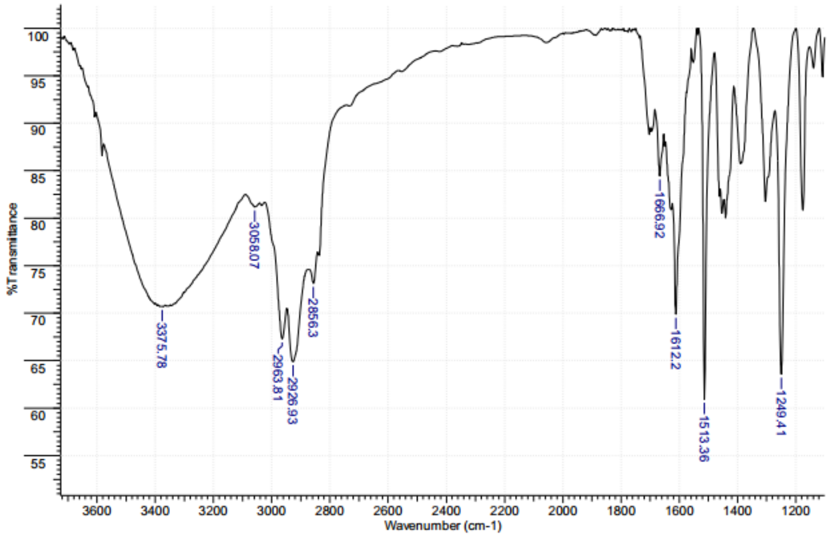

Figure S14. IR spectrum of compound **8**.

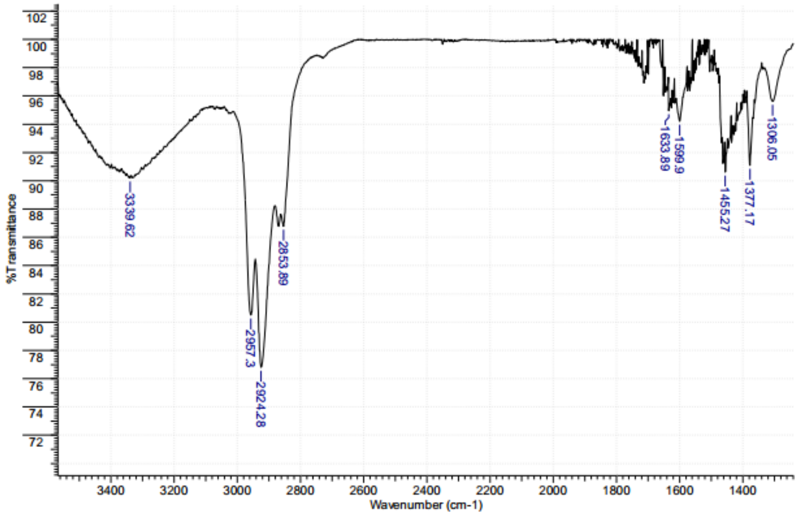

Figure S15. IR spectrum of compound **9**.

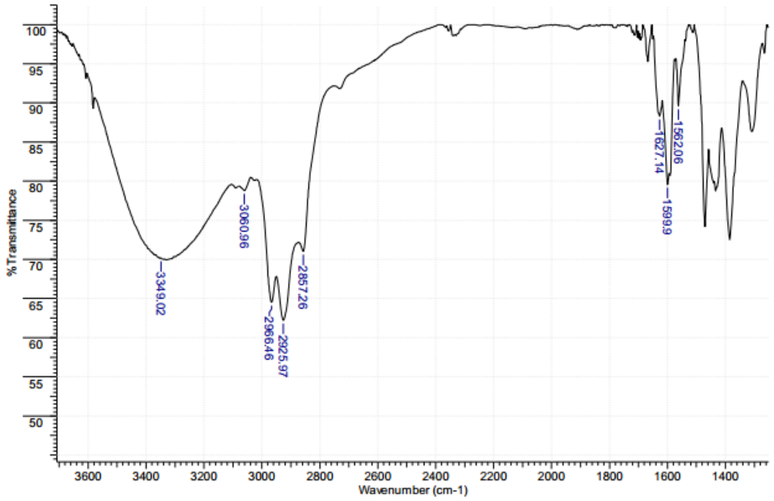

Figure S16. IR spectrum of compound **10**.

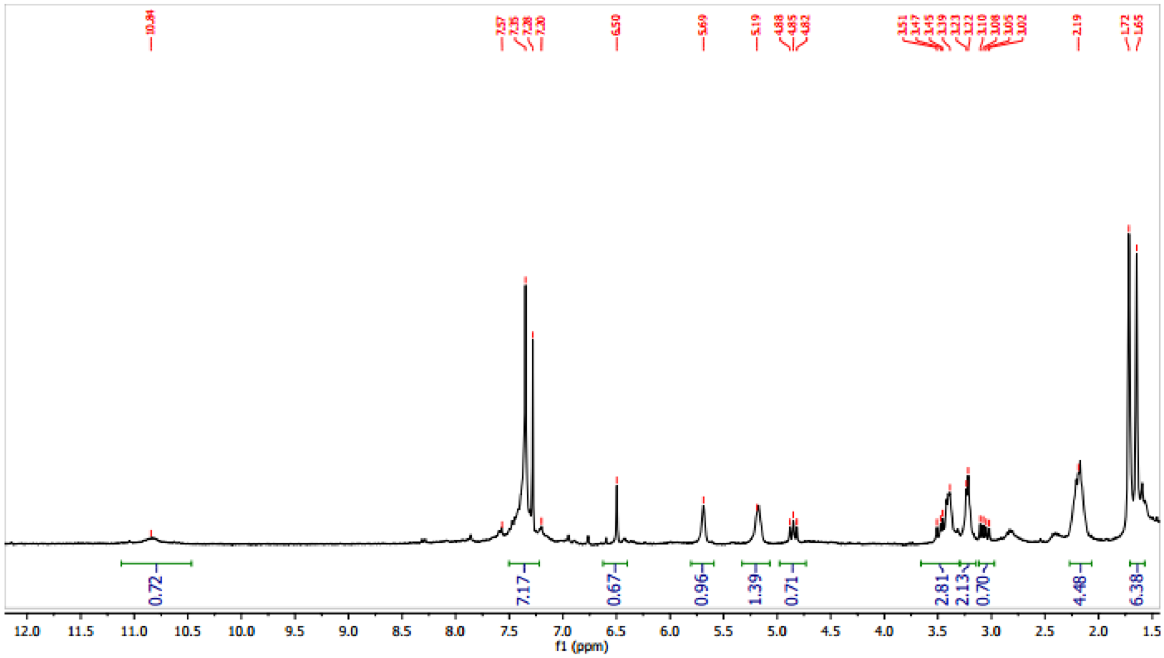

Figure S17. ^{1}H NMR spectrum of compound **7**.

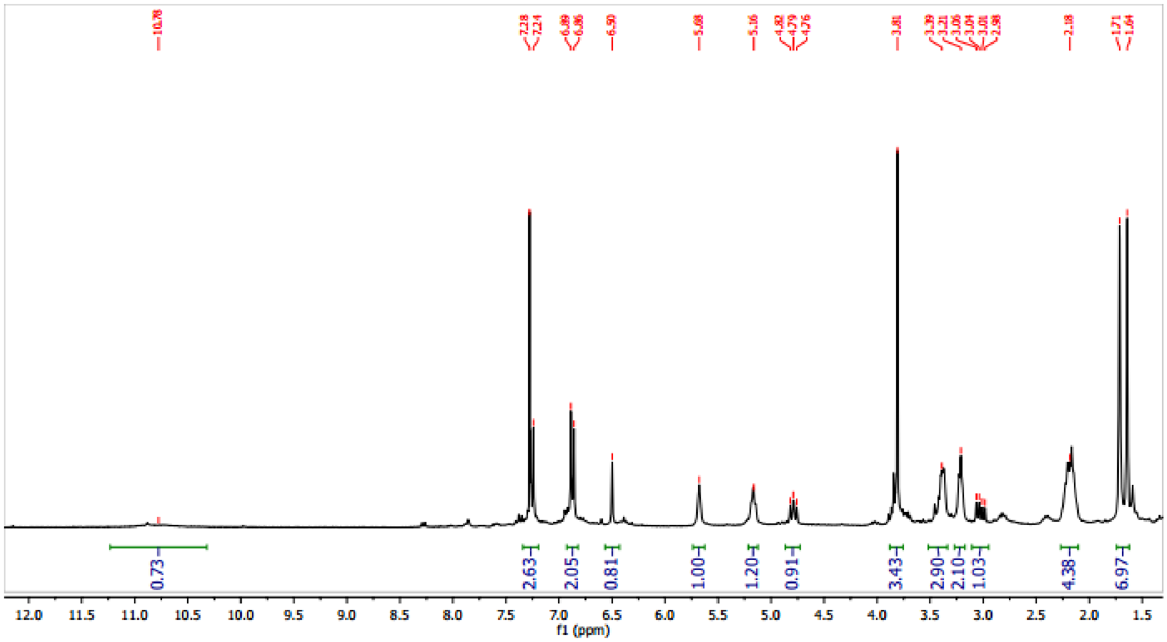

Figure S18. ^1H NMR spectrum of compound **8**.

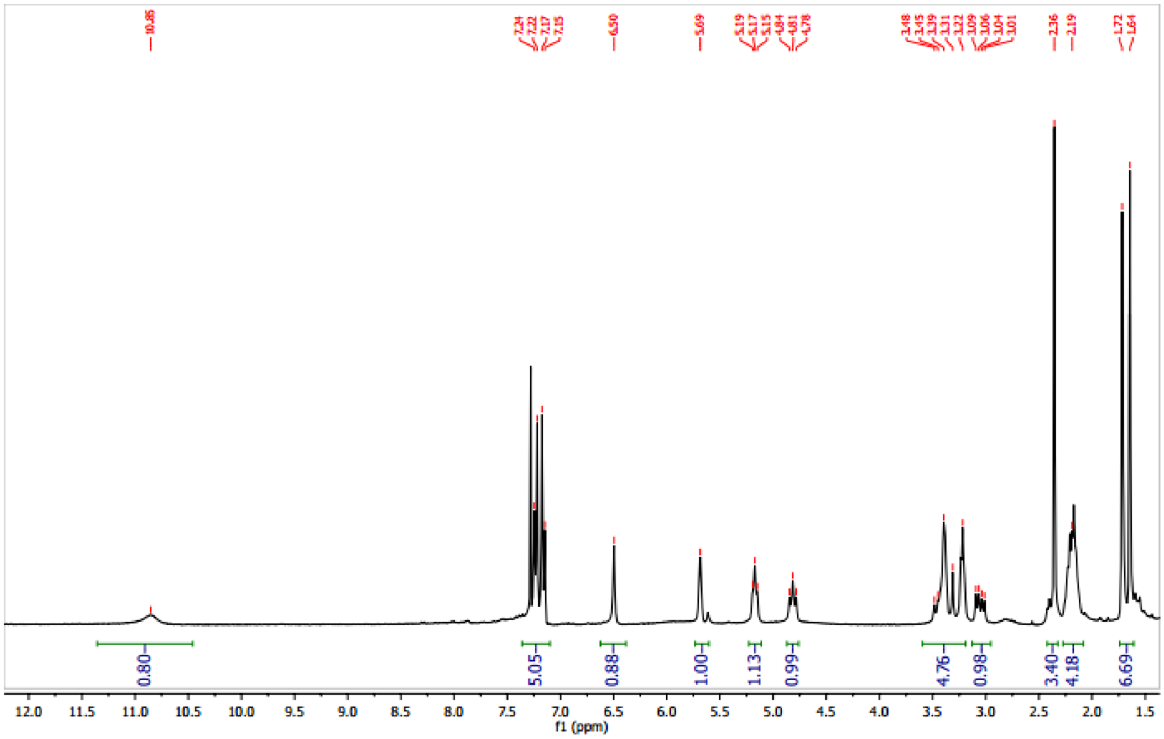

Figure S19. ^{1}H NMR spectrum of compound **9**.

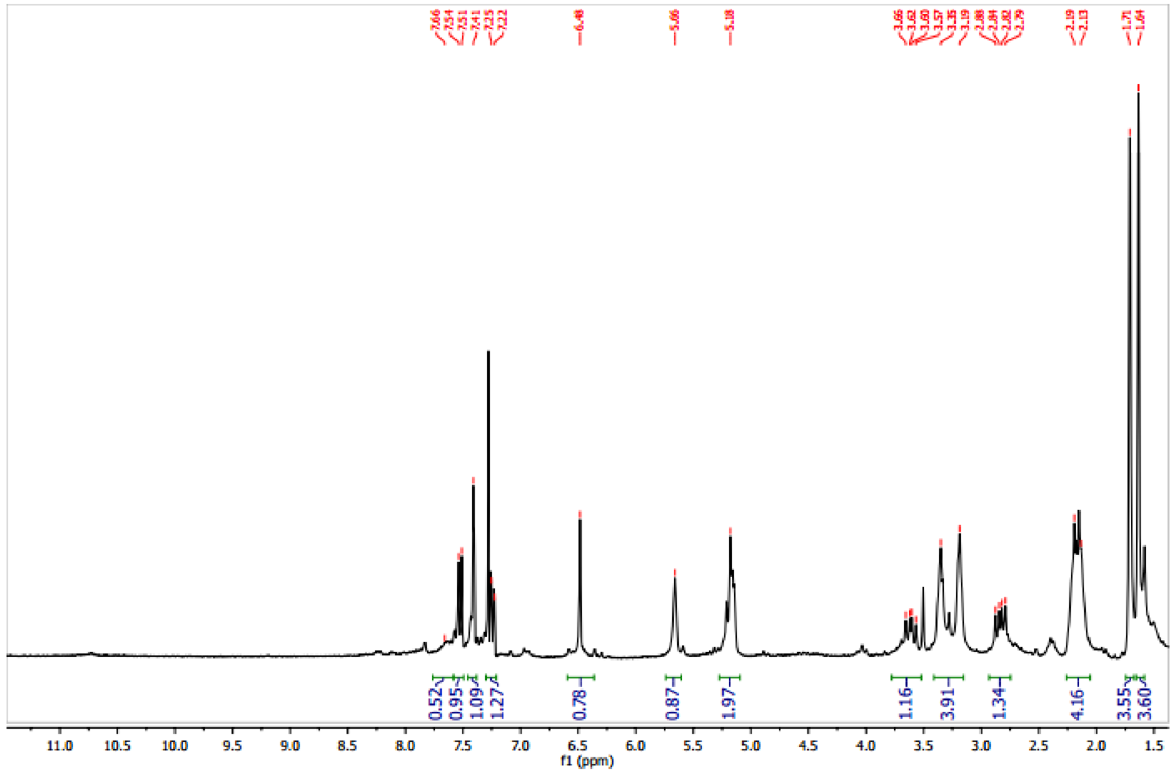

Figure S20. ^{1}H NMR spectrum of compound **10**.

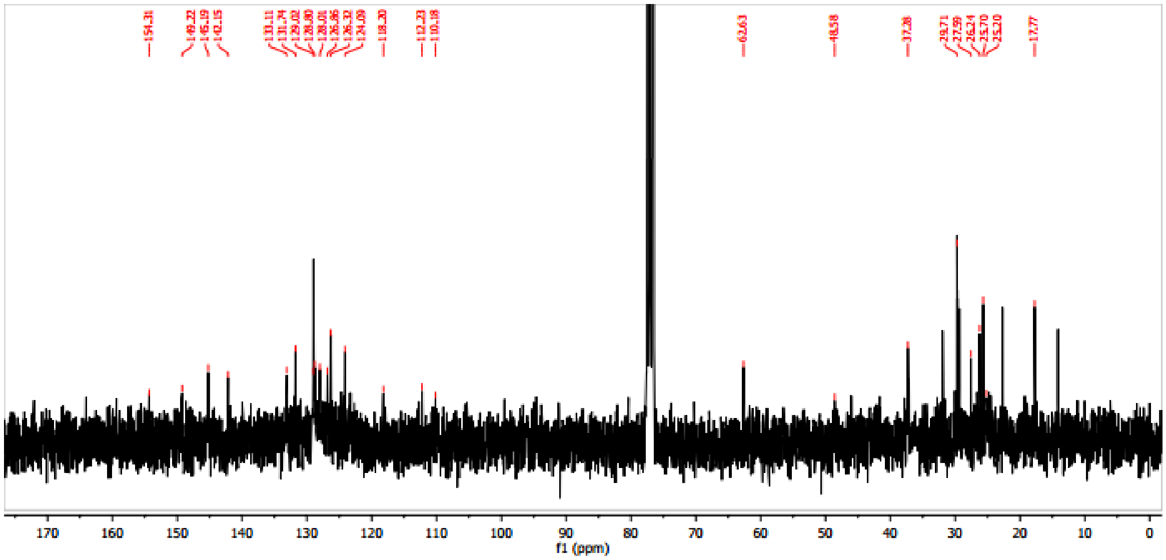

Figure S21. ^{13}C NMR spectrum of compound **7**.

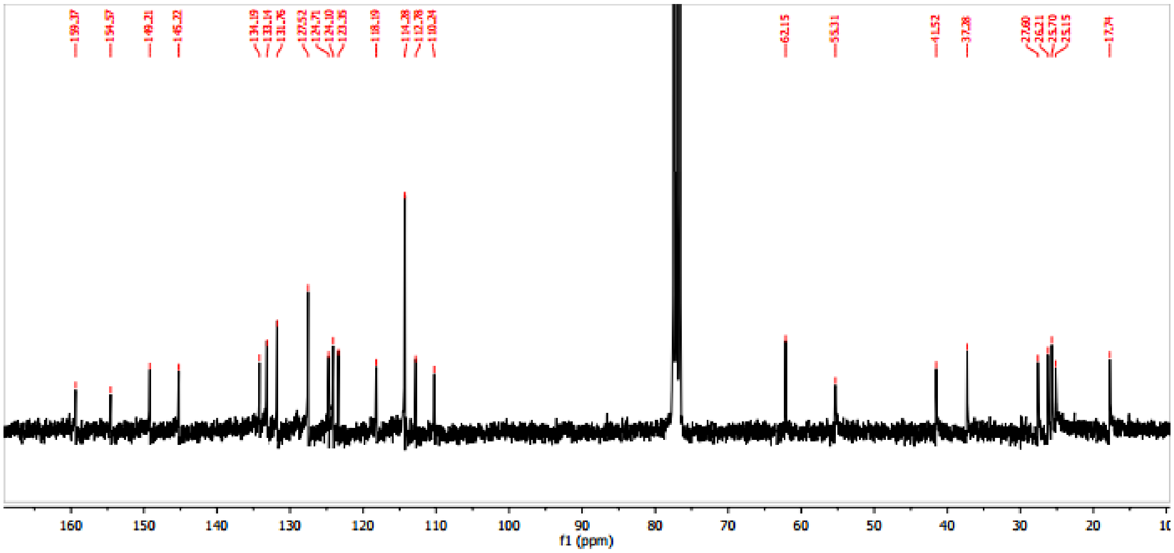

Figure S22. ^{13}C NMR spectrum of compound **8**.

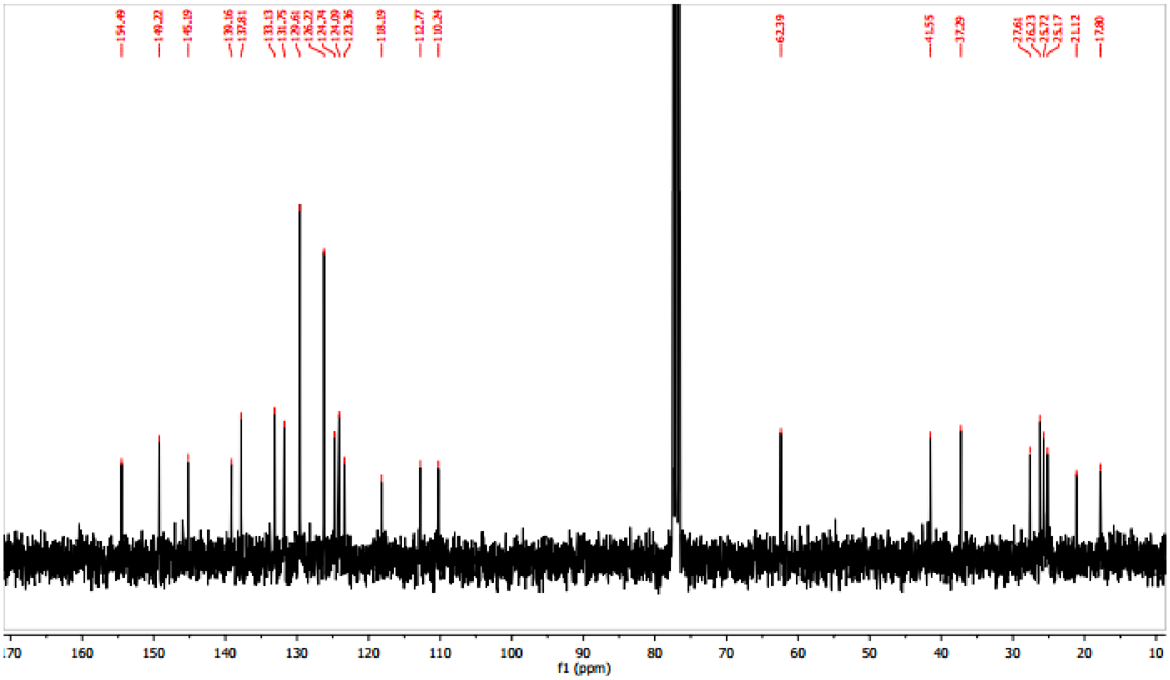

Figure S23. ^{13}C NMR spectrum of compound **9**.

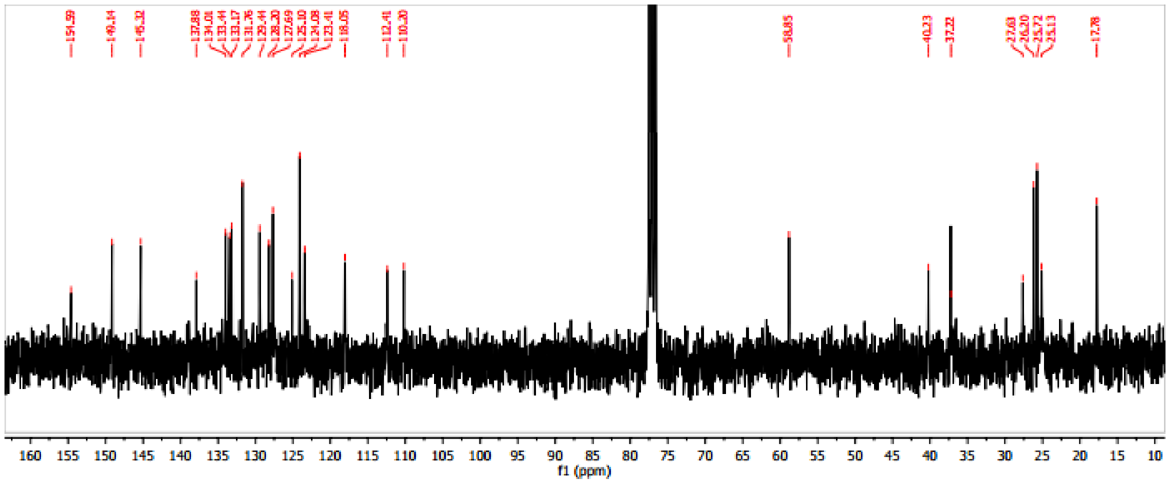

Figure S24. ^{13}C NMR spectrum of compound **10**.

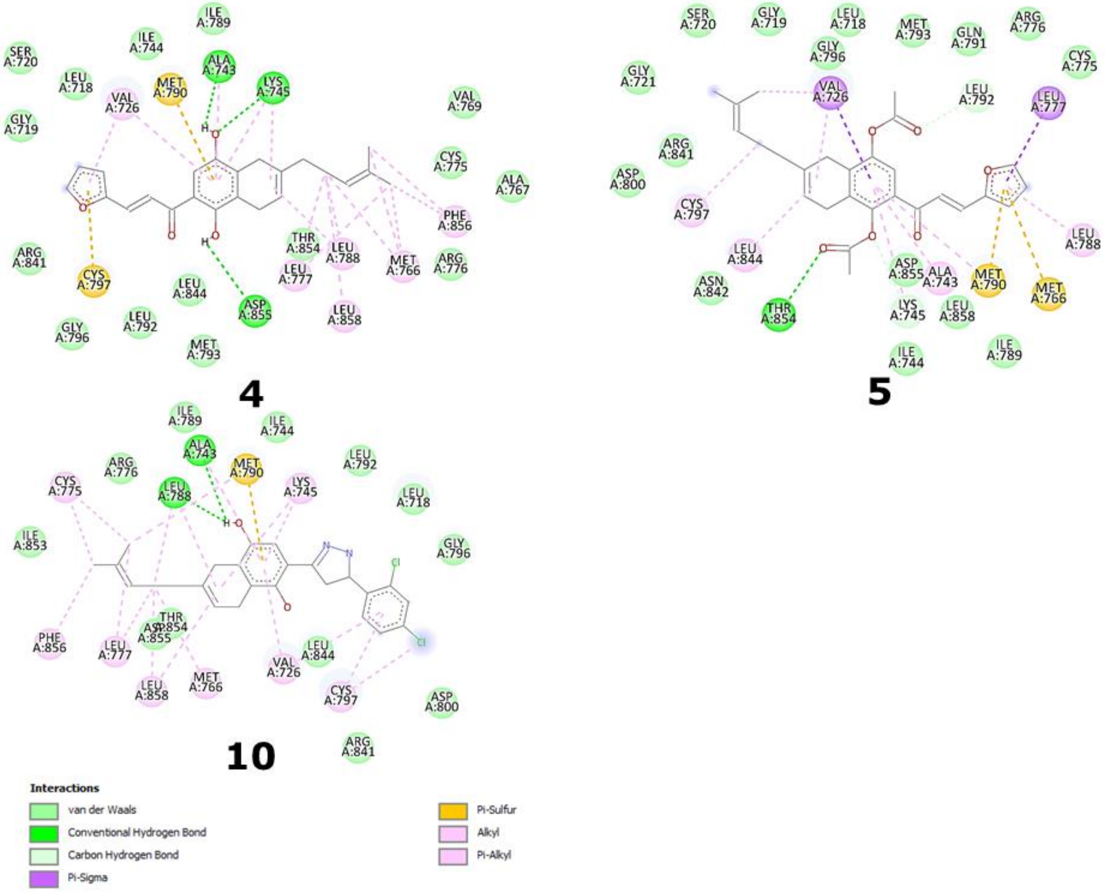

Figure S25. Plotted 2D maps of H-bonds and hydrophobic interactions of CHBQs 4 and 5, and PIBHQ 10 with EGFR residues.

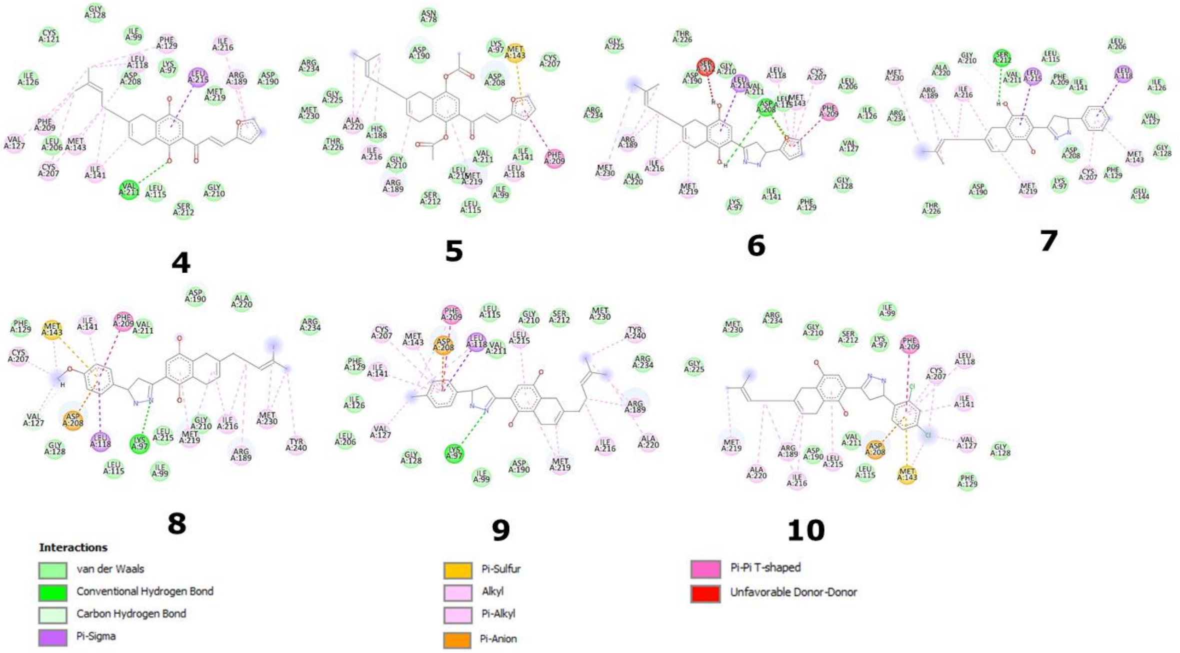

Figure S26. Plotted 2D maps of H-bonds and hydrophobic interactions of CHBQs **4** and **5**, and PIBHQs **6–10** with MEK1 residues.

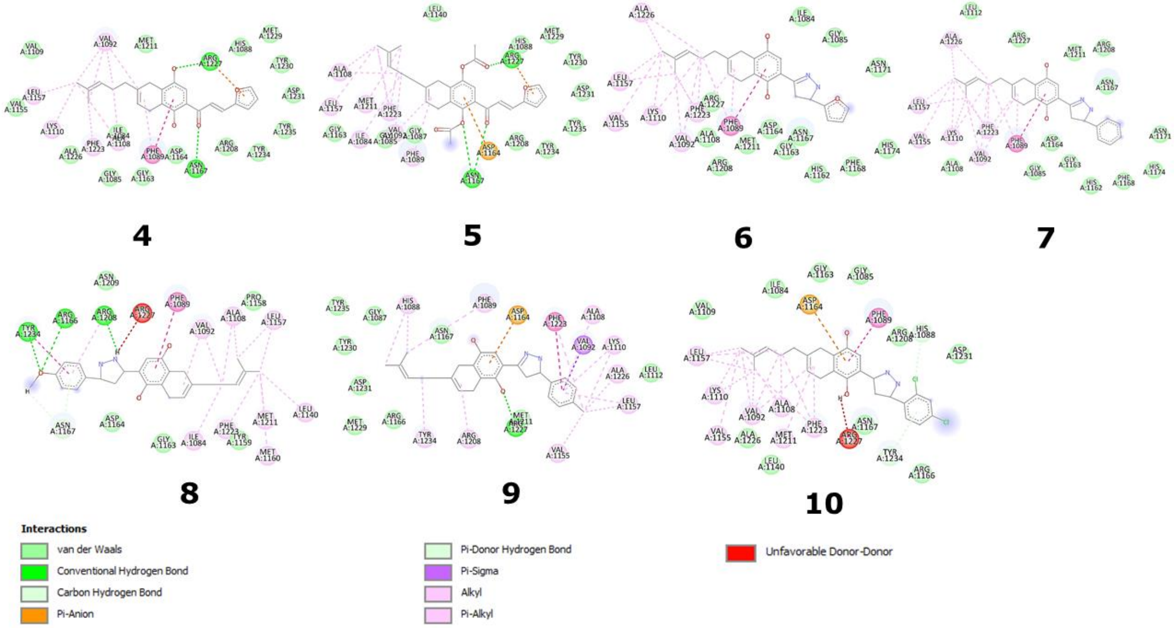

Figure S27. Plotted 2D maps of H-bonds and hydrophobic interactions of CHBQs **4** and **5**, and PIBHQs **6–10** with c-MET residues.

Table S1. Predicted binding free energy values (ΔGbin kcal/mol) of chalcone/pyrazoline-1,4-benzohydroquinone hybrids with selected proteins overexpressed in cancer.

Compounds	Target proteins																Total avge.
	**DHFR**	**COX-2**	**FGFR-2**	**VEGRF-2**	**NR3A1**	**EGFR**	**HER2**	**NR3A2**	**c-MET**	**TRKA**	**ERK2**	**MEK1**	**CK4**	**TPK**	**TopoII**	**TUB**	
**4**	-9.1	-9.2	-8.9	-9.9	-8.3	-10.7	-7.8	-9.0	-9.7	-9.2	-9.2	-9.6	-8.4	-8.6	-8.3	-8.7	-9.11
**5**	-7.2	-8.3	-8.7	-7.4	-8.3	-8.5	-7.6	-6.9	-9.5	-9.2	-8.8	-8.5	-8.3	-8.5	-7.5	-8.6	-8.26
**6**	-8.7	-9-0	-9.1	-10.2	-8.7	-11.3	-9.1	-9.4	-10.3	-9.5	-9.9	-10.0	-7.9	-10.0	-8.6	-9.0	-9.55
**7**	-9.5	-8.6	-9.6	-10.2	-8.8	-11.4	-9.0	-10.0	-9.8	-10.0	-9.5	-10.7	-8.7	-10.4	-8.5	-9.5	-9.82
**8**	-9.5	-8.5	-9.3	-8.7	-8.5	-11.0	-7.8	-9.9	-9.7	-9.3	-9.5	-10.3	-8.8	-10.2	-8.5	-9.2	-9.42
**9**	-9.5	-8.9	-9.7	-10	-8.9	-11.2	-9.2	-10.3	-9.9	-10.1	-9.9	-10.6	-9.0	-10.6	-9.8	-9.6	-9.92
**10**	-9.6	-8.7	-9.8	-9.9	-8.8	-11.4	-8.5	-9.9	-9.9	-9.9	-9.9	-10.4	-9,2	-10.6	-9.4	-8.8	-9.83
**P avge.**	-9.01	-8.74	-9.30	-9.47	-8.61	**-10.79**	-8.43	-9.34	**-9.83**	-9.60	-9.53	**-10.01**	-8.61	**-9.84**	-8.66	-9.06	
**Doxorubicin**	-7.7	-8.3	-9.0	-9.1	-7.7	-8.9	-6.2	-8.2	-8.7	-9.0	-9.2	-9.1	-7.4	-10.1	-7.7	-8.1	

Proteins with their respective (PDB) entries: **DHFR**: Dihydrofolate reductase (1DLS); **COX-2**: Cyclooxygenase 2 (3LN2); **FGFR-2**: Fibroblast growth factor receptor 2 (1GJO); **VEGRF-2**: Vascular endothelial growth factor receptor 2 (3VHE); **NR3A1**: Estrogen receptor α (3ERT); **EGFR**: Epidermal growth factor receptor (5GTY); **HER2**: Epidermal growth factor receptor 2 (7JXH); **TRKA**: Tropomyosin receptor kinase A (6PL2); **NR3A2**: Estrogen receptor beta (2QTU); **c-MET**: Mesenchymal-epithelial transition factor (3RHK); **TRKA**: Tropomyosin receptor kinase A (6PL2); **ERK2**: Extra-cellular signal-regulated kinase 2 (2OJG); **MEK1**: MAPK/ERK kinase (4AN3); **CK4**: Cyclin-dependent kinase 4 (1PXL); **TPK**: Tyrosine-protein kinase (4EHZ); TopoII: Topoisomerase II (5GWK); **TUB**: alpha tubulin (6WSL). P avge.: Protein average. mean of the ΔG_{bin} values for the interactions of each protein with all the hybrids; the two proteins with the highest global chalcone/pyrazoline-1,4-benzohydroquinone affinity are highlighted on red color.

Table S2. Predicted binding free energy values (ΔGbin, kcal/mol) and binding site contacts of synthesized cytotoxic hybrids with amino acids of TPK, TRKA and CK4.

Compounds	ΔGbin	H-Bonds and Hydrophobic Contacts in the Binding Site*
		**TPK** (mean ΔG*bin* = -9.72 kcal/mol)
**4**	-8.9	Leu881, Gly882, Glu883, **Gly884**, His885, Phe886, Gly887, Lys888, Val889* Ala906, Lys908 *, Met956*, **Asp1003**, **Arg1007**, Asn1008, Leu1010, Gly1020, Asp1021 *, Gly1023, Leu1024, Asp1042
**5**	-8.5	Gly882, Glu883, Gly884, His885, Phe886, Gly887, Lys888, Val889, Lys908, Leu910, His918, Asp921, Leu922, Lys924, Glu925, Arg1002, Asp1003, Arg1007, Asn1008, Gly1020, Asp1021, Gly1023, Leu1024 *, Lys1026, Val1037, Asp1039*, Asp1042, Pro1044
**6**	-10.0	Leu881, Gly882, Glu883, **Gly884**, His885*, Phe886*, Gly887, Lys888, Val889, Ala906, Lys908*, Leu910, His918, Asp921, Leu922, Met956, Ser963, Asp1003, **Arg1007**, **Asn1008**, Leu1010, Gly1020, Asp1021*, Gly1023, Leu1024
**7**	-10.4	Leu881, Gly882, Glu883, Gly884, His885, Phe886, **Gly887**, Lys888, Val889, Ala906, Lys908*, Leu910, His918, Asp921, Leu922, Glu925, Met956, Ser963, **Asp1003**, **Arg1007**, Asn1008, Leu1010, Gly1020, **Asp1021***, Gly1023, Leu1024
**8**	-10.2	Leu881, Gly882, Glu883, Gly884, His885, Phe886*, **Gly887**, Lys888, Val889, Ala906, Lys908*, His918, Met956, Ser963, Arg1002, **Asp1003**, Arg1007, Asn1008, Leu1010, Gly1020, Asp1021*, Leu1024, Asp1039, Asp1042
**9**	-10.6	Leu881*, Gly882, Glu883, Gly884, His885, Phe886, Gly887, Lys888, Val889*, Ala906, Lys908*, Leu910, His918, Asp921, Leu922, Phe958, Leu959, Gly962, Ser963, Glu966, Asp1003, **Arg1007**, **Asn1008**, Leu1010*, Asp1021*, Gly1023, Leu1024
		**TRKA** (mean ΔGbin = -9.55 kcal/mol)
**4**	-9.2	Leu516*, Gly517, Val524*, Ala542, Lys544, Glu560, Leu564*, Ile572, Val573*, Phe589, **Glu590**, Tyr591, Met592, Gly595, Asp596, Arg599, Leu657, Arg673, Ile666, Gly667, Asp668 *, Phe669
**5**	-9.2	Leu516, Gly517, Glu518, Gly519, Val524*, Ala542, Lys544, Glu560, Leu564*, Val573*, Phe589*, Glu590, Tyr591, Met592, Gly595, Asp596, Leu657, Gly667, Asp668, Phe669*, Arg673, Ile675, Tyr676
**6**	-9.5	Leu516, Gly517, Glu518, Val524, Ala542, Lys544, Glu560, Glu564, Val573, Phe589, Glu590, Tyr591, Met592, Gly595, **Asp596**, Leu657, Gly667, Asp668, Phe669*, **Arg673**, Ile675*, Tyr676
**7**	-10.0	Leu516, Gly517, Val524, Ala542, Lys544, Glu560, Leu564, Val573, Phe589, Glu590, Tyr591, Gly595, **Asp596**, Arg599, Leu657, Leu667, Asp668, Ile675*, Arg673, Tyr676, Asp668, Phe669*
**8**	-9.3	Leu516, Gly517, Val524*, Ala542*, Lys544, Glu560, Leu564*, Val573*, Phe589*, Tyr591, Met592, His594, Gly595, Asp596, Arg599, Leu657, Gly667, Asp668, Phe669 *, Arg673
**9**	-10.1	Leu516*, Val524, Ala542, Lys544, Glu560, Leu564, Val573*, Phe589*, Tyr591, Gly595, Asp596, Arg599, Leu657*, Gly667, Asp668, Phe669 *, Arg673, Ile675, Tyr676
		**CK4** (mean ΔG*bin* = -8.52 kcal/mol)
**4**	-8.4	Ile10, Gly11, Glu12, Gly13, Thr14, Val18, Lys33, Phe82, Leu83, His84, Gln85, Asp86, Lys89, Lys129, **Gln131**, **Asn132**, Leu134, Ala144, Asp145, Glu162, Val163, Val164
**5**	-8.3	Glu8, Ile10, Gly11, **Glu12**, Gly13, Thr14, Val18, Lys20, Ala31, **Lys33**, Phe82, Leu83, His84, Gln85, Asp86*, Lys88, Lys89, Lys129*, Gln131, **Asn132**, Leu134, Ala144, Asp145, Leu298
**6**	-7.9	**Ile10**, Gly11, Glu12, Glu13, Thr14, Tyr15, Val18, Lys33, Phe82, Leu83, His84, Gln85, Asp86, Lys89, Asp127, Lys129, Gln131, **Asn132***, Leu134, **Asp145**
**7**	-8.7	Ile10*, Gly11, Glu12, Gly13, Thr14, Val18, Lys20, Lys33*, Phe82, **Leu83**, His84, Gln85, Asp86, Lys89, Lys129, Gln131, Asn132, Leu134, Ala144, Asp145, Thr160, Glu162, Val163, Val164
**8**	-8.8	Glu8, Ile10*, Gly11, Glu12, Gly13, Thr14, Val18, Lys20, Lys33*, Phe82, **Leu83**, His84, Gln85, Asp86, Lys89, Lys129, Gln131, Asn132, Leu134, Ala144, **Asp145**, Thr160, Glu162, Val163, Val164
**9**	-9.0	Glu8, Ile10*, Gly11, Glu12, Gly13, Thr14, Val18, Lys20, Lys33, Phe82, **Leu83**, His84, Gln85, Asp86, Lys89, Lys129, Gln131, Asn132, Leu134, Ala144, **Asp145**, Thr160, Glu162, Val163, Val164

Bolded names correspond to those amino acids involved in H-bonds with the corresponding CBHQs and PIBHQs.
Residues with * correspond to amino acids that interact with the ligand by any type of Pi-interactions
